# Integrated multi-omics analysis of single-cell and spatial transcriptomics reveals distinct hpv-associated immune microenvironment features and prognostic signatures in cervical cancer

**DOI:** 10.3389/fimmu.2025.1612623

**Published:** 2025-09-16

**Authors:** Qiuyue Su, Xiangdong Tian, Fucheng Li, Xi Yu, Wenchen Gong, Yurong Chen, Jianan Wang, Siqi Yang, Shaojun Zhang, Qian Zhang, Shanshan Yang

**Affiliations:** ^1^ Department of Gynecological Radiotherapy, Harbin Medical University Cancer Hospital, Harbin, China; ^2^ Department of Endoscopy, Tianjin Medical University Cancer Institute & Hospital, National Clinical Research Center for Cancer, Tianjin’s Clinical Research Center for Cancer, Tianjin Key Laboratory of Digestive Cancer, Tianjin, China; ^3^ Department of Breast Surgery, Harbin Medical University Cancer Hospital, Harbin, China; ^4^ Medical Research Institute, Guangdong Provincial People’s Hospital (Guangdong Academy of Medical Sciences), Southern Medical University, Guangzhou, China; ^5^ Department of Abdominal Radiotherapy, Harbin Medical University Cancer Hospital, Harbin, China

**Keywords:** cervical cancer, HPV, single-cell sequencing, spatial transcriptomics, tumor microenvironment, prognostic factor

## Abstract

**Background:**

Cervical cancer (CC) is a highly heterogeneous malignancy primarily driven by persistent infection with high-risk human papillomavirus (HPV). However, comprehensive analyses of heterogeneity in the immune microenvironment, particularly its spatial heterogeneity, between HPV-positive and HPV-negative CC remain limited, despite their critical clinical significance.

**Methods:**

We performed single-cell RNA sequencing (scRNA-seq) and spatial transcriptomics (ST) sequencing on collected cervical cancer samples, integrating scRNA-seq, ST, and bulk RNA-seq to analyze distinct cell subtypes and characterize their spatial distribution. Multiplex immunofluorescence analysis was further utilized to validate HPV status-specific expression patterns. Cox regression and LASSO regression analyses were used to identify the prognostic signature on the TCGA dataset.

**Results:**

Through integrative analysis, we found that HPV-positive samples demonstrated elevated proportions of CD4^+^ T cells and cDC2s, whereas HPV-negative samples exhibited increased CD8^+^ T cell infiltration. In HPV-positive CC, epithelial cells acted as primary regulators of cDC2s via the *ANXA1*-*FPR1/3* pathway, with cDC2s subsequently modulating CD4^+^ T cells and interferon-related CD8^+^ T cell subtypes. In contrast, HPV-negative CC featured epithelial cells predominantly influencing monocytes and macrophages, which then interacted with CD8^+^ T cells. Notably, the *MDK*-*LRP1* ligand-receptor interaction emerged as a potential key mechanism for recruiting immunosuppressive cells into CC tumors, fostering an immunosuppressive microenvironment. Further, we constructed a risk score model based on an epithelial cell-related signature (ERS), which was significantly associated with patient survival. Noteworthy variations were observed in immune cell infiltration and immune microenvironment among distinct risk groups.

**Conclusion:**

Based on integrated multi-omics data, we precisely delineated the spatial transcriptional features of the tumor microenvironment in CC with different HPV statuses, including identifying distinct CD8^+^ T cell states and cell-cell communication. In addition, we developed an ERS closely associated with the immune environment and prognosis of CC. These results increase our understanding of the molecular mechanisms of cervical cancer under different HPV statuses and provide assistance for the precise treatment of cervical cancer.

## Introduction

Cervical cancer (CC) remains one of the most lethal malignancies affecting women worldwide (1), with persistent infection of high-risk human papillomavirus (HPV) being the primary etiological factor. Epidemiological evidence indicates that high-risk HPV infection accounts for over 90% of CC cases (2, 3). Compared to HPV-positive patients, HPV-negative patients exhibit higher rates of metastasis and increased mortality in advanced stages (3, 4). However, the underlying mechanisms responsible for these differences remain poorly understood, and metastatic CC patients (especially HPV-negative patients) have difficulty receiving effective clinical treatment.

The critical influence of the tumor microenvironment (TME) on carcinogenesis and therapeutic effectiveness has been widely recognized. Cancer cells can adapt to and thrive in novel microenvironments by modulating their interactions with stromal cells, altering their mechanical properties (5), and regulating key signaling pathways, all of which contribute to their survival and migration potential (6). These findings suggest that the heterogeneity of the TME, especially in tumor epithelial cells, is crucial for determining the malignant phenotype of cancer cells. However, the molecular mechanisms by which different TME components affect immunotherapy efficacy remain unclear in CC, as does the link between the poor prognosis in HPV-negative patients and the TME.

Single-cell sequencing technology plays a crucial role in studying the TME in CC, enabling a detailed understanding of the cellular composition and interactions that drive tumor progression and response to therapies (7). Currently, several studies based on single-cell transcriptomic sequencing data have explored the components of the TME and the cell-cell interactions within TME. However, the interactions between cells are inherently dependent on their spatial proximity, and single-cell sequencing lacks spatial information, which limits the understanding of how these interactions occur in their native tissue context. The emergence of spatial transcriptomics (ST) has addressed this limitation by combining the high precision of RNA sequencing with spatial resolution, constructing detailed transcriptional and spatial maps to probe transcriptional activity at the spatial level (8). Integrating ST and single-cell transcriptomics allows for a more detailed evaluation of the TME in HPV-negative and HPV-positive samples, which is crucial for elucidating the genomic and molecular differences based on HPV infection status and may provide important insights for developing more targeted and effective treatment strategies.This study presents scRNA-seq or ST analysis of 7 biopsy samples each from untreated donors, categorized as either HPV-negative or HPV-positive CC. This comprehensive approach aims to elucidate the features of distinct cell subpopulations and identify dynamic changes occurring in both the tumor and microenvironment, with a specific focus on HPV infection status. We revealed heterogeneity within CC tumors by mapping super-resolution spatial maps of CC, identified key cell interactions that lead to the immunosuppressive microenvironment, and verified these findings through ST and multiplex immunofluorescence (mIF). Based on epithelial cells, the source of differences in cell communication, we constructed a prognostic signature through the epithelial cell-related signature (ERS), which has good potential in predicting the prognosis of CC patients and assessing immunotherapy response. Overall, these findings underscore the molecular heterogeneity within the TME based on the HPV infection status and highlight different potential targets for future cancer therapy.

## Materials and methods

### Patient sample collection

Four fresh samples of pathologically diagnosed squamous cell carcinoma of the cervix were collected from Harbin Medical University Cancer Hospital, with written informed consent obtained from all patients and approval from the Institute Research Medical Ethics Committee of Harbin Medical University (Ethics Approval No. KY2019-16). Conducted in accordance with the Declaration of Helsinki, the study included two HPV-positive and two HPV-negative CC individuals for scRNA-seq analysis.

HPV infection status was determined using a commercial HPV Genotyping Diagnosis Kit (Genetel Pharmaceuticals, Shenzhen, China) with parallel analysis via HPV genotype DNA microarray reader system (HPV-GenoCam-9600, Genetel Pharmaceuticals), in addition, P16 expression was evaluated through immunohistochemistry. Detailed clinical and pathological information is presented in **Supplementary Table S1**. Fresh tumor samples acquired prior concurrent chemoradiotherapy, and no patient received chemotherapy, radiotherapy, or immunotherapy before. After washing with phosphate-buffered saline (PBS), the specimens were finely minced into pieces smaller than 1 mm^3^ using a scalpel on ice and placed in 1 mL of Cryopreservation Protection Fluid (SINOTECH™ Tissue Sample Cryopreservation Kit, JZ-SC-58202, Sinomics Genomics, China). Initially frozen at -80 °C overnight in a gradient freezer, the samples were then transferred to liquid nitrogen for long-term storage, facilitating subsequent scRNA-seq and analysis.

### Single‐cell sequencing analysis

All procedures adhered to the manufacturer’s protocol (BD Biosciences). Single-cell suspensions from each sample were initially stained with two fluorescent dyes, Calcein AM (Thermo Fisher Scientific, Cat. No. C1430) and Draq7 (Cat. No. 564904), to accurately determine cell concentration and viability using the BD Rhapsody™ Scanner before proceeding with single-cell multiplexing labeling. Cell viability ranged from 70% to 80%. Each sample’s single-cell suspension was sequentially labeled with the BD Human Single-Cell Multiplexing Kit (Cat. No. 633781) before pooling. The BD Rhapsody Express system, using a micro-well cartridge, captured the single-cell transcriptome. Approximately 18–000 cells were captured across more than 200–000 micro-wells in each batch. Excess oligonucleotide barcode beads were loaded onto the cartridge to ensure nearly every micro-well contained one bead paired with a micro-well. Cells were lysed with cell lysis buffer, releasing polyadenylated RNA molecules that hybridized with the beads. The beads were then harvested into a single tube for reverse transcription. During cDNA synthesis, each cDNA molecule was labeled with a molecular index and a cell label indicating its source cell at the 3’ end of the mRNA transcript. The entire transcriptome library was prepared through double-strand cDNA synthesis, ligation, and general amplification involving 13 PCR cycles. To enrich the 3’ end of transcriptional products associated with cell labels and molecular indices, the sequencing library of the whole transcriptome amplification products was prepared using random start-up PCR. These libraries were sequenced on the HiSeq2500 (Illumina) using the PE150 model. The BD Whole Transcriptome Analysis (WTA) Rhapsody analysis pipeline was used to process sequencing data, including alignment and generation of gene/barcode matrix.

### 10x Genomics Visium ST

The tumor and background tissue sections from another 3 patients were processed for ST using the 10x Genomics Visium platform. Visium spatial gene expression data for formalin-fixed paraffin-embedded (FFPE) tissues were acquired in strict accordance with the meticulously detailed protocols furnished by 10× Genomics for tissue preparation and library construction. The FFPE tissues underwent a sequential series of processing steps. First, they underwent deparaffinization, a process essential for removing paraffin wax. This was followed by staining, which facilitated the visualization and identification of tissue components. Subsequently, the version 1 human whole-transcriptome probe panels were applied to the deparaffinized, stained, and decrosslinked tissues. Upon completion of the hybridization process, where complementary nucleic acid sequences anneal to each other, ligation of the probes was carried out. The ligation products were liberated from the tissue through RNase treatment and permeabilization techniques. Spatially barcoded oligonucleotides were then utilized to capture the ligated probe products, and an extension reaction of the probes was subsequently initiated. Libraries were generated from the extended probes through standard molecular biology techniques such as polymerase chain reaction-based amplification and purification steps. To assess the integrity and quantity of each resultant library, a Qubit fluorometer and an Agilent TapeStation were employed, leveraging their respective capabilities for accurate nucleic acid quantification and quality assessment. The final libraries were subjected to high-throughput sequencing on an Illumina NovaSeq 6000 platform. This sequencing process yielded 28-base-pair (bp) reads, which encompassed spatial barcodes and unique molecular identifier (UMI) sequences. The spatial barcodes were crucial for mapping the origin of each sequenced fragment within the tissue, while the UMIs enabled the quantification of unique transcripts, reducing the impact of PCR amplification biases. In addition, 50-bp probe reads were generated, which were essential for transcriptomic profiling and gene expression analysis.

### ST analysis

The gene-spot matrices generated after processing the ST data of ST and Visium samples were analyzed using the Seurat package (version 4.3.0). Spots were filtered based on a minimum detected gene count of 200 genes, and genes with fewer than 10 read counts or expressed in fewer than 3 spots were removed. Inter-spot normalization was performed using the LogVMR function. Dimensionality reduction and clustering were carried out on the first 30 principal components using principal component analysis. To better visualize the spatial expression of features, the spots were enhanced using the “spatialEnhance” function of the BayesSpace package (version 1.6.0) (9). The “enhanceFeatures” was utilized to enhance and calculate the horizontal expression of all genes of interest. We performed an integrated analysis of scRNA-seq data and ST data using the CellTrek R package (10) to calculate the spatial k-distances between different cell subsets and specific cell subsets.

### Inferring super-resolution tissue architecture

The cell type composition of each point was determined using the iStar algorithm. The iStar is a weakly supervised model (11). It trains the model on multiple two-dimensional ST section data and annotates the super-resolution tissue structure of the sections through machine learning. Firstly, iStar adopts self-supervised learning to pre-train a hierarchical vision transformer on the available tissue staining datasets, achieving the effect of hierarchical layer feature extraction. Subsequently, iStar uses weakly supervised learning to train a feed-forward network model, which predicts the ultra-high-pixel-level gene expression map using the previously obtained features. Finally, this model divides the gene expression measurement at each point in ST sequencing into multiple values and assigns them to each super-pixel. By integrating the feature information in the histological images, it predicts the gene expression at the super-pixel level.

### Raw sequencing data processing and identification of major cell populations

Quality control and downstream analysis of the scRNA-seq-derived gene expression matrix were performed using the Seurat pipeline (v4.3.0) in R (v4.2.3) (12). Cells with fewer than 800 genes with nonzero counts were excluded. Additionally, cells where over 25% of the counts originated from the mitochondrial genome were filtered out as low quality. After rigorous quality control, a total of 8843 single cells were retained. The filtered expression matrix was normalized using Seurat’s standard workflow to generate normalized count data.

Highly variable genes were selected for unsupervised clustering analysis through the Seurat pipeline. Dimensionality reduction was achieved using the first 20 principal components derived from 2000 highly variable genes. Cell clustering was performed with the FindClusters function at a resolution of 0.8, followed by two-dimensional visualization using Uniform Manifold Approximation and Projection (UMAP) (13). Differentially expressed genes (DEGs) within the same cell clusters across different groups and marker genes for each cluster were calculated using the ‘FindAllMarkers’ function with default thresholds. A manual review process was then conducted to identify major cell types based on the enrichment of specific markers within each cell cluster.

### Calculation of the gene set activity level

Gene set activity analysis was performed on TCGA-CESC(Cervical Squamous Cell Carcinoma and Endocervical Adenocarcinoma) samples using the AUCell R package (version 1.20.2) (14). The analytical workflow comprised three key steps (1): Computing gene expression rankings for each sample using the AUCell_buildRankings function with default parameters (2); Scoring predefined cellular gene sets (identified via Seurat’s FindAllMarkers function with default thresholds) against these rankings; and (3) Quantifying gene set enrichment through Area-under-the-curve (AUC) values using the AUCell_calcAUC function, which reflects the fraction of top-expressed genes belonging to each gene set. The TCGA cohort was stratified by HPV status (positive vs. negative), followed by comparative analysis of AUC values between subgroups.

### Pseudotime trajectory analysis

Developmental trajectories of heterogeneous cell clusters were reconstructed using Monocle2 (version 2.26.0) (15), with cluster-specific marker genes identified by Seurat’s FindAllMarkers function (default parameters) serving as input features. Focusing on T cell immunobiology, we specifically analyzed differentiation trajectories of CD8^+^ and CD4^+^ T cell subsets by (1): Projecting cellular states into a reduced-dimensional space using DDRTree (2); Ordering cells along pseudotemporal axes to infer differentiation pathways; and (3) Applying BEAM analysis to identify branch point-associated DEGs. This computational framework enabled systematic characterization of potential lineage commitment events within T cell populations across experimental groups.

### Cell-cell communication analysis

Intercellular communication networks were systematically analyzed using CellChat (version 1.6.1) to delineate HPV status-dependent signaling patterns (16). Differential ligand-receptor (L-R) interactions were quantified through the netVisual_bubble function ‘s comparison parameter, with dominant sender-receiver cell pairs visualized via 2D scatter plots. Key signaling hubs were identified by (1): Specifying source/target cell populations; and (2) Generating strength-ranked bar charts of top interactions. The netVisual_diffInteraction function quantified epithelial cells-specific communication disparities with monocytes and cDC2s between groups. Complementary analysis was performed using CellPhoneDB Python (version 4.0.0) (17), a Python-based repository of curated L-R interactions, retaining significant pairs (p<0.05). The iTALK platform further characterized communication networks, with functional annotation of L-R pairs through its integrated database and Circos plot visualization emphasizing immunosuppressive checkpoint interactions.

### MHC II and DC maturation scores

Gene signature scores were computed at single-cell resolution using Seurat’s AddModuleScore function. Two specific immunological signatures were quantified (1): MHC class II score, derived from the expression profile of MHC class II-related genes; and (2) Dendritic cell (DC) maturation score, calculated based on established DC maturation markers (**Supplementary Table S2**). These scores represent the average expression levels of respective gene sets normalized against control gene features.

### Differential gene expression analysis

Differential gene expression analysis was performed to compare: (i) HPV-infected versus non-infected epithelial cells, and (ii) HPV-infected versus non-infected cDC2s, using Seurat’s FindMarkers function with Wilcoxon rank sum test. Significant DEGs were selected based on the following criteria (1): log fold change (lnFC) > 0.25 (2), p < 0.05, and (3) minimum percentage (min.pct) > 0.1. Pathway enrichment analysis of epithelial cell-specific DEGs was subsequently conducted using the ReactomePA package (version 1.42.0) through its enrichPathway function, with false discovery rate (FDR) correction for multiple testing.

### Establishment and verification of prognostic model

Utilizing epithelial cell differential genes with prognostic value, an ERS was established to predict the prognosis of CC. Firstly, univariate Cox regression analysis was employed to evaluate the impact of these genes on the survival status of CC patients. Subsequently, the least absolute shrinkage and selection operator (LASSO) regression method was utilized to further reduce the number of candidate genes, ultimately constructing the ERS. With the ERS, patients were classified into a high-risk group or a low-risk group according to the median risk score. Receiver operating characteristic (ROC) curves, calibration curves, and decision curve analysis (DCA) were applied to assess the accuracy and clinical utility of the ERS.

### Differences in the immune TME

The “CIBERSORT” R package (18) was used to strictly assess the differences in the immune TME within different risk groups and clarify the complex differences in immune cell infiltration among different risk groups. The Ro/e (Ratio of observed to expected) analysis was used to assess the distribution preferences of cell subsets in different tissues (19). The “estimate” R package (20) was used to quantify Stromal Score, Immune Score and EATIMATE Score in patients with CC.

### Multiplex immunofluorescence

mIF was carried out using a multiple fluorescent staining kit (abs50014; Absin, Shanghai, China) according to the manufacturer’s instructions. Briefly, the sections were dewaxed in xylene, rehydrated through an alcohol gradient, and incubated with serum. Following heat repair, the sections were incubated with primary antibodies (*CD8*, *CD4*, *PD1*, Zhongshan Chemical Co., Beijing, China; *ANXA1*, *MDK*, BIoss; Beijing, China) and then with secondary antibodies conjugated to 520, 570, 690, 650 or 620. *DAPI* was subsequently used to stain the nuclei. The sections were then examined under a laser scanning confocal microscope (Zeiss, Pleasanton, CA, USA).

## Results

### Super-resolution tissue architecture in HPV-positive and HPV-negative CC

**Figure 1:**
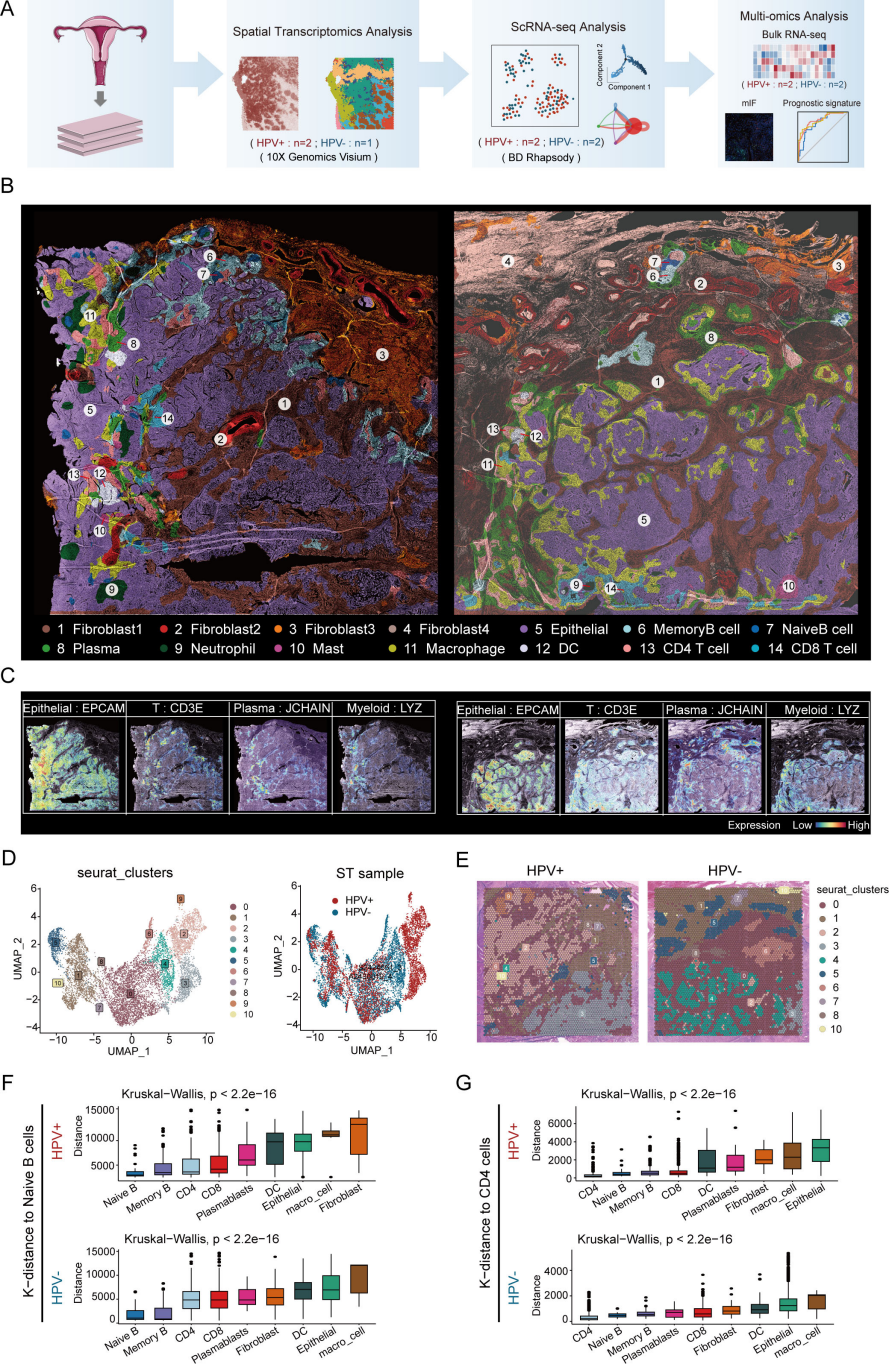
Super-resolution tissue architecture resolved by iStar in HPV-positive and HPV-negative CC. **(A)** Flow chart. **(B)** High-resolution spatial clustering and representative gene expression enhanced with iStar in HPV- positive CC (left) and HPV- negative CC (right)(Dark brown represents fibroblast1, deep red represents fibroblast2, orange represents fibroblast3, light brown represents fibroblast4, purple represents epithelial cell, azure represents memory B cell, dark blue represents naive B cell, light green represents plasma cell, dark green represents neutrophil, purplish red represents mast cell, yellow represents macrophage, white represents DC, pink represents CD4^+^ T cell, and light blue represents CD8^+^ T cell). **(C)** ST analysis depicting the expression profiles of various marker genes, the left panel illustrates the distribution of epithelial cells (*EPCAM*), T cells (*CD3E*), plasma cells (*JCHAIN*), and myeloid cells (*LYZ*) in an HPV-positive sample; the right panel shows the expression of these markers in an HPV-negative sample. The color gradient represents the level of expression, ranging from low (blue) to high (red). **(D)** Visualizing the distribution of cell clusters in HPV-positive and HPV-negative samples by UMAP plot. **(E)** Spatial distribution of various cell clusters in HPV-positive and HPV-negative samples. **(F)** CellTrek calculated the average k-distance from different cell types to naive B cells. **(G)** CellTrek calculated the average k-distance from different cell types to CD4^+^ T cells.

To explore TME heterogeneity between HPV-positive and HPV-negative CC, we performed ST with tumor sections from three patients with CC (**Figure 1A**). The currently widely used 10X Visium platform covers more than 25 cells per spot and thus cannot obtain information at single-cell resolution on the tissue. Based on previous studies, gene expression patterns were correlated with histological image features, suggesting the possibility of predicting gene expression from histology. Therefore, using algorithms to mine information from hematoxylin and eosin images and ST data to reconstruct super-resolution tissue images becomes a possibility (21, 22). Utilizing iStar (11), we constructed near-single-cell resolution spatial architecture maps of CC tissue on the histological images for HPV - positive(left) and HPV-negative (right) patients (**Figure 1B**). By employing distinct gene markers, we visualized the spatial distribution of epithelial cells (*EPCAM*), T cells (*CD3E*), plasma cells (*JCHAIN*), and myeloid cells (*LYZ*), revealing their organization within the TME (**Figure 1C**). The spatial distribution of gene markers of major cell types precisely coincides with the locations where these cells exist, which has been confirmed by two pathologists with reference to the original histological sections.

Based on cellular activities and molecular characteristics, we identified four different fibroblast subtypes. Of note, one of which was present only in HPV-negative patients (fibroblast 4). This discrepancy may be due to a series of cellular and molecular changes caused by HPV infection, which in turn affect the differentiation and existence status of fibroblasts. Meanwhile, we identified the spatial locations of nine immune cell subtypes, including three B cell subtypes (memory B cell, naive B cell and plasma), four myeloid cell subtypes (neutrophil, mast, macrophage and DC), and two T cell subtypes (CD4^+^ T cell and CD8^+^ T cell). These immune cells are present in both tumor and stromal regions, which is consistent with the findings of previous scRNA-seq studies (23).

We performed UMAP visualization of cell clusters using Seurat and analyzed the marker gene expression in each cluster (**Figures 1D, E**; **Supplementary Figures S1A, B**). It was important to note that Seurat clustering reflects similarities in the overall gene expression patterns within spots (55 μm) rather than the clustering of individual cell types. Thus, the cell clusters in UMAP indirectly corresponded to regions with similar cell type compositions (**Figure 1E**).

Our findings indicated that the distribution of cell clusters identified by Seurat clustering was similar to the distribution of cell types determined by the iStar method. For example, spots dominated by epithelial cells in Seurat clusters (clusters 2, 3, 4, 6, and 9) exhibited a similar distribution in tissue sections to the epithelial cell subsets identified by the iStar method; spots dominated by immune cells in the Seurat cluster (cluster 0) showed a similar distribution to the T cell subsets identified by the iStar method. These results further validated the authenticity and superiority of the iStar method, as it can identify different cell types within each spot as accurately as possible by integrating histological images.

In addition, we found that the spatial locations of memory B cells and naive B cells are adjacent, and the spatial locations of CD4^+^ T cells and DCs are also adjacent, whether in HPV-positive and HPV-negative patients. We validated the results of iStar by calculating the average k-distance from various cell types to specific cells (naive B cells and CD4^+^ T cells) in each tissue section using the CellTrek spatial analysis method. The results showed that in both HPV-positive and HPV-negative patients, memory B cells were spatially closest to naive B cells, while DCs were relatively close to CD4^+^ T cells (**Figures 1F, G**), which was consistent with the results of iStar. This spatial proximity phenomenon may be related to the development and differentiation of B cells (24), antigen presentation and immunological activation (25), and the coordinated interaction among immune cells (26, 27). In summary, we showed the distribution of various cell types and the expression patterns of classic markers in HPV-positive and HPV-negative patient tissues at high resolution spatial images, providing detailed cellular and molecular information in TME of CC.

### Single-cell landscape in HPV-positive and HPV-negative CC

**Figure 2:**
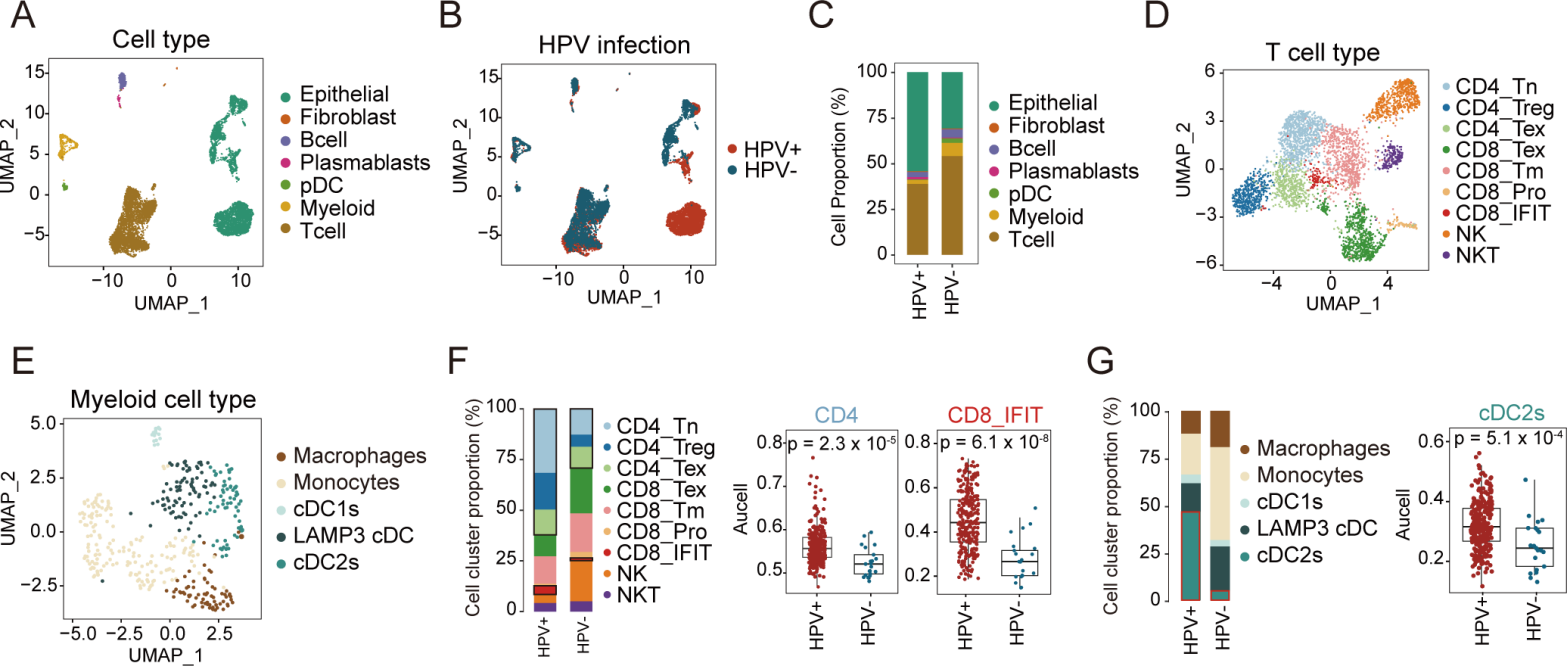
Single-cell landscape of HPV-positive and HPV-negative CC. **(A, B)** UMAP map of 8,843 cells profiled, with each cell color coded for: seven indicated cell types and tissue types. **(C)** Proportion of each cell type between HPV-positive and HPV-negative samples. **(D)** UMAP plots of T and NK cell subsets, colored by cell types. **(E)** UMAP plots of myeloid cells, colored by cell types. **(F)** Proportion of each cell type in T and NK cells between HPV-positive and HPV-negative samples (left); the comparison of AUCell scores for CD4, CD8_IFIT in HPV-positive and HPV-negative samples (right, Wilcoxon test). **(G)** Proportion of each cell type in Myeloid cells between HPV-positive and HPV-negative samples (left); the comparison of AUCell scores for cDC2s in HPV-positive and HPV-negative samples (right, Wilcoxon test). UMAP: Uniform Manifold Approximation and Projection. pDC, plasmacytoid dendritic cell.

We conducted single-cell RNA sequencing (scRNA-seq) on samples derived from untreated CC patients, stratified into HPV-negative and HPV-positive cohorts (**Figure 1A**). After quality control, a total of 8,843 high-quality cells were retained for downstream analysis. Using cell type-specific markers, we identified seven major cell subpopulations: epithelial cells (3,889 cells, 44%; marked by *EPCAM*, *KRT19*, *CD24*, *CDH1*), T cells (4,011 cells, 45.3%; marked by *CD3D*, *CD3E*), B cells (315 cells, 3.6%; marked by *CD19*), plasmablasts (78 cells, 0.9%; marked by *MS4A1*), fibroblasts (41 cells, 0.5%; marked by *COL1A1*, *DCN*, *C1R*), plasmacytoid dendritic cells (pDCs, 116 cells, 1.3%; marked by *LILRA4*, *XCR3*, *IRF7*), and myeloid cells (393 cells, 4.4%; marked by *CD68*, *LYZ*, *TYROBP*) (**Figure 2A**; **Supplementary Figure S2A, B**). No cell type was observed to exclusively exist in a specific HPV status, although some cell types were more abundant in either HPV-positive or negative samples (**Figure 2B**). Specifically, compared to HPV-positive samples, HPV-negative samples exhibited a relatively lower proportion of epithelial cells, while the proportions of T cells were relatively higher (**Figure 2C**; **Supplementary Table S3**). Given the limited size of our sample cohort, tissue preference of each cluster was validated by Ro/e analysis to adjust cell-sampling biases for each patient. (**Supplementary Figure S2C**). This suggests that in HPV-negative patients, immune cells are more extensively recruited to the tumor tissue, potentially participating in anti-tumor immune responses (**Figure 2C**).

T and myeloid cells critically regulate tumor progression in the TME, where T cells particularly mediate antiviral and antitumor immunity (28–31). To understand the role of T and myeloid cells in HPV infection and tumor development, we further divided T and myeloid into different subtypes based on cell marker expression (**Figures 2D, E**; **Supplementary Figures S2D, H**; **Supplementary Table S4**). T cells were classified into nine subtypes, comprising three CD4^+^ T cell subtypes (naïve CD4^+^ T, regulatory T cells (Treg), and exhausted CD4^+^ T), four CD8^+^ T cell subtypes (exhausted CD8^+^ T, memory CD8^+^ T, proliferating CD8^+^ T, and interferon-related CD8^+^ T), and two NK cell subtypes (NKT and NK) (**Supplementary Figure S2D**),. Myeloid cells were divided into five subtypes: macrophages, monocytes, LAMP3+ DCs, cDC1s, and cDC2s (**Supplementary Figure S2H**).

Our analysis of T cell subtypes revealed distinct patterns between HPV- and HPV+ samples. Among CD4^+^ T cells, all three subtypes showed lower proportions in HPV- samples. Conversely, in CD8^+^ T cell subtypes, except for CD8^+^ IFIT, other CD8^+^ T cell subtypes were present at higher proportions in HPV- samples (**Figure 2F**, left; **Supplementary Figures S2E, F, G**). These findings suggest differential immune dynamics between HPV- and HPV+ samples. Consistent results were observed in our analysis of the TCGA cohort, where HPV- patients exhibited lower infiltration of both CD4^+^ T cells and CD8^+^ IFIT compared to HPV+ patients (**Figures 2F**, right).

In parallel, we analyzed the myeloid compartment to explore potential differences in immune cell composition. In HPV- patients, we observed higher proportions of macrophages, monocytes, and LAMP3+ DCs, while cDC2s were significantly reduced compared to HPV+ patients (**Figure 2G**, left). Analysis of the TCGA cohort corroborated these results, revealing significantly higher infiltration of cDC2s in HPV+ patients compared to HPV- patients (p = 5.1 × 10^−4^; **Figure 2G**, right; **Supplementary Figure S2I**). Together, these findings suggest that HPV+ patients may exhibit a stronger immune response mediated by cDC2s and CD4^+^ T cells, whereas HPV- patients may rely more on CD8^+^ T cell-mediated immunity.

### Distinct CD8^+^ T cell trajectories in HPV-positive versus HPV-negative

**Figure 3:**
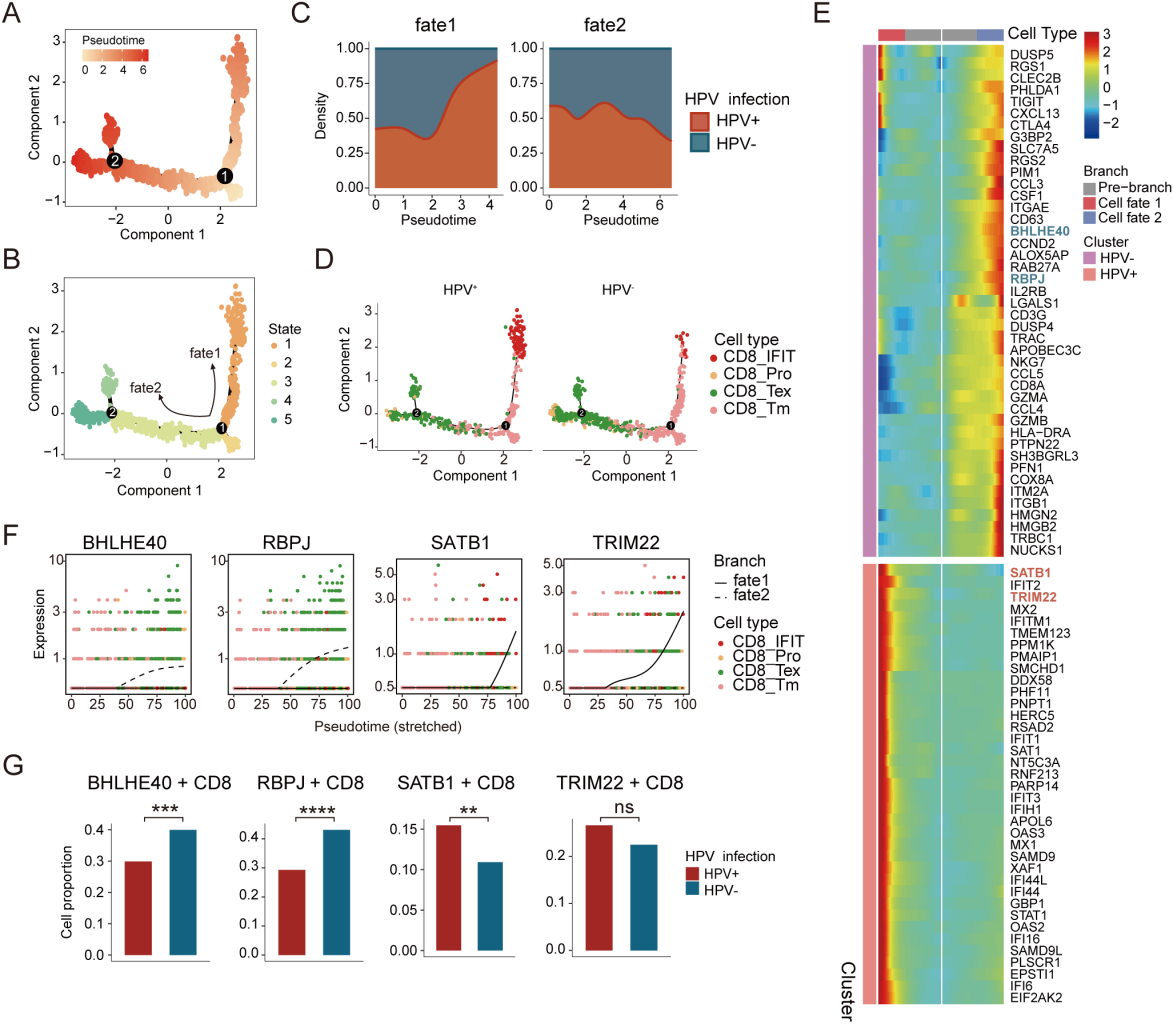
Distinct developmental trajectory inference between HPV-positive and HPV-negative CC. **(A)** Monocle analysis for trajectory inference of CD8^+^ T-cell subpopulations during the transition, colored by pseudotime. **(B)** Monocle analysis for trajectory inference of CD8^+^ T-cell subpopulations during the transition, colored by states. **(C)** Cell density distribution reflecting the relative number of CD8^+^ T cells along the CD8^+^ trajectories between HPV-positive and HPV-negative samples. **(D)** Distribution of CD8^+^ T-cell subpopulations of HPV-positive (left) and HPV-negative (right) during the transition along the cell types. **(E)** Heatmap showing the dynamic differences in DEGs of distinct branches (The blue font indicate genes highly expressed along the fate 2 trajectory, while the red font indicate genes highly expressed within the fate 1 trajectory). **(F)** Cell types and expression of representative genes along the pseudotime for CD8^+^ T cells. **(G)** Expression proportion of the BHLHE40, RBPJ, SATB1, and TRIM22 genes between HPV-positive and HPV-negative CD8^+^ T cells (chi-square test, *p < 0.05, **p < 0.01, ***p < 0.001, ****p < 0.0001).

T cells play a central role in the TME and constitute the predominant immune cell population (32). The analysis of cellular composition in HPV-negative and HPV-positive patients demonstrated differences in the distribution of T cell subtypes. However, comparative trajectory analysis revealed no significant differences in CD4^+^ T cell differentiation between the two groups (**Supplementary Figures S3A, B**). CD8^+^ T cells, known for their cytotoxic properties, play critical roles in tumor suppression and viral infection control (33). Monocle 2 analysis of all CD8^+^ T cells constructed a pseudotemporal trajectory that identified five distinct cellular states, which segregated into two divergent branches (**Figures 3A, B**). Notably, we observed a progressive increase in HPV+ cells in fate 1, whereas fate 2 was predominantly enriched for HPV− cells (**Figure 3C**). Combining pseudotime trajectory with CD8^+^ T cell subtypes, we defined memory CD8^+^ T cells as the root state. Along Fate 1, CD8^+^ Tm cells progressively transitioned toward interferon-responsive CD8^+^ T cells (CD8^+^ *IFIT*). In contrast, Fate 2 bifurcated into two terminal states: exhausted CD8^+^ T cells and proliferative CD8^+^ T cells (**Figure 3D**). This divergence suggests distinct functional reprogramming paths driven by HPV infection status.

We further analyzed transcription factors (TFs) dynamically regulated along the pseudotime trajectory (**Figure 3E**). In Fate 2, the expression of *BHLHE40* and *RBPJ* progressively increased (**Figure 3F**), with higher levels observed in HPV− compared to HPV+ cells (**Figure 3G**). *BHLHE40* is increasingly recognized as a key regulator of immunity in infection, autoimmunity, and inflammation, acting as a critical checkpoint between progenitor and effector T cell subsets (34, 35). *RBPJ*, a mediator of Notch signaling, plays a pivotal role in immune cell development and differentiation and is negatively correlated with CD8^+^ T cell cytotoxic function (36, 37). Their upregulation in Fate 2 suggests a Notch-dependent differentiation bias toward exhaustion and impaired effector potential in HPV− patients.

Conversely, Fate 1 exhibited increasing expression of *STAB1* and *TRIM22* (**Figure 3F**). Stabilin-1 (*STAB1*), a scavenger receptor linked to cellular trafficking, inflammation, and cancer, exerts protective anti-infective effects by modulating cytokine/chemokine production and immune cell recruitment (38). *TRIM22*, an interferon-stimulated gene, mediates antiviral responses and immune regulation (39). Their enrichment in Fate 1 implies that HPV infection may trigger interferon-driven autoimmunity, accompanied by *STAB1*/*TRIM22* upregulation (**Figure 3G**). Together, these results demonstrate that HPV infection status drives divergent CD8^+^ T cell differentiation programs - an interferon-dominated antiviral response (*STAB1*/*TRIM22*) in HPV+ patients versus Notch-mediated exhaustion (*BHLHE40*/*RBPJ*) in HPV- patients.

### HPV+ and HPV- cervical cancers diverge in TME cell-cell communication

**Figure 4:**
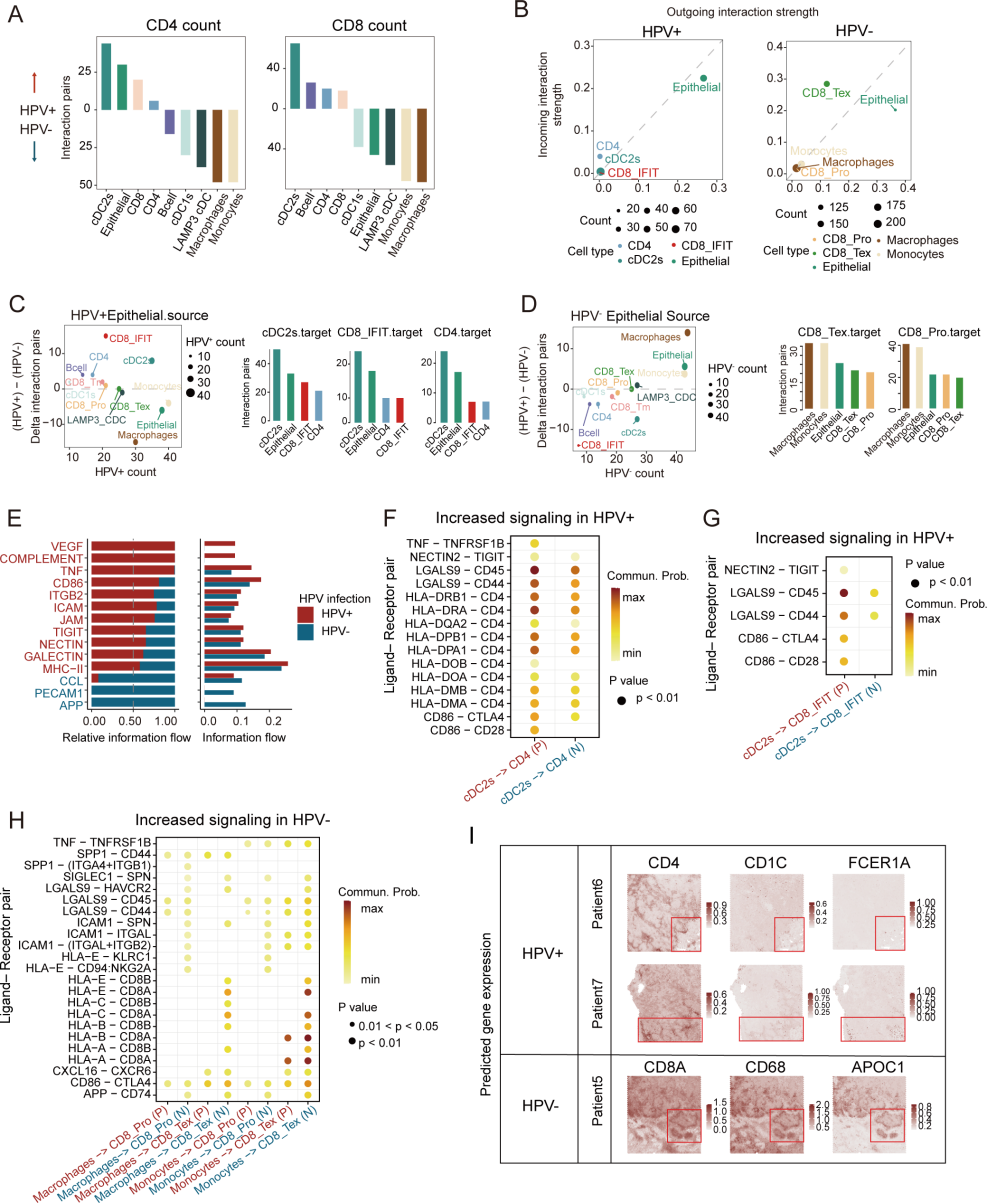
Characteristics of cell-cell communication between HPV-positive and HPV-negative CC in TME. **(A)** Bar plots depicting interaction intensities between major cell types and CD4^+^ (left)/CD8^+^ (right) T cells. Upward bars represent stronger interactions in HPV-positive CC, while downward bars indicate stronger interactions in HPV-negative CC, colored by cell types. **(B)** Cell clusters located based on the count of their significant incoming (Y-axis) or outgoing (X-axis) signaling pattern in HPV-positive samples (left) and HPV-negative samples (right). **(C)** Left: Differential signaling patterns originating from HPV^+^ vs. HPV^−^ epithelial cells (Y-axis: HPV^+^ − HPV^−^; X-axis: HPV^+^ signaling count). Right: Bar plot illustrating the count values of signaling transmission among primary cell types in HPV-positive samples. **(D)** Left: Differential signaling patterns originating from HPV^−^ vs. HPV^+^ epithelial cells (Y-axis: HPV^−^ − HPV^+^; X-axis: HPV^−^ signaling count). Right: Bar plot illustrating the count values of signaling transmission among primary cell types in HPV-negative samples (HPV^+^, HPV-positive; HPV^−^, HPV-negative). **(E)** Bar plot depict relative (left) and absolute (right) information flow of ligand-receptor pathways from cDC2s to CD4^+^ T cells, with HPV-positive (red) and HPV-negative (blue) signaling patterns compared along the X-axis. **(F–H)** Bubble plot comparing significant ligand-receptor pairs in HPV-positive and HPV-negative samples, illustrating signaling interactions from cDC2s to CD4 cells **(F)**, from cDC2s to CD8_IFIT cells **(G)**, and from macrophage/monocyte cells to CD8_Tex/CD8_Pro cells **(H)**. The color of the points reflects the communication probability, while the size of the points represents the computed p-value. Blank areas indicate zero communication probability. (P, HPV-positive; N, HPV-negative) **(I)** Enhanced spatial feature plots showing the expression of *FCER1A*, *CD1C*, *CD4*, *CD68*, *APOC1*, and *CD8A* in HPV-positive and HPV-negative CC tumor tissues.

To delineate HPV-specific crosstalk patterns, we performed CellphoneDB and Cellchat analyses comparing CD4^+^/CD8^+^ T cell interactions. In HPV+ tumors, CD4^+^/CD8^+^ T cells preferentially communicated with cDC2 cells, whereas HPV- tumors exhibited enhanced T cell-macrophages/monocytes interactions (**Figure 4A**). Epithelial cells emerged as the dominant signal senders in both HPV- and HPV+ tumors, with the highest ligand-receptor outgoing strength, while CD8^+^ T cells functioned as central signal integrators through robust incoming interactions (**Figure 4B**). Quantitative interaction weight scores further validated these patterns (**Figures 4C, D**), revealing an HPV-dependent rewiring of the CC immunoregulatory network with therapeutic implications.

Analysis of DC/T-cells transitions revealed activated VEGF and complement signaling pathways in HPV-positive CC, whereas the APP signaling pathway was more prominent in HPV-negative CC (**Figure 4E**). Previous studies have shown that VEGF signaling upregulation occurs upon T-reg cells depletion in lung adenocarcinomas. Virus-derived factors like VEGF can recruit immunosuppressive cells to establish a pro-viral microenvironment (40). Notably, cDC2-CD4^+^ cell communication showed enriched MHC II and CD4 signaling in HPV+ tumors (**Figure 4F**). DEG analysis identified elevated DC maturation genes (*SERPINB9*, *NR4A3*, *PRDM1*, etc.) in HPV-positive cDC2s, versus upregulated suppression markers (*LILRB2*, *TNFAIP3*) in HPV-negative tumors (**Supplementary Figure S3D**). HPV-positive tumors also exhibited higher MHC II and DC maturation scores (**Supplementary Figure S3E**). As cDC2s excel in the cell polarization and antigen presentation (41), their predominant interaction with interferon-responsive CD8^+^ T cells (*LGALS9*-*CD45*) (**Figure 4G**) may enhance anti-tumor immunity. HPV-positive cDC2s were specifically enriched in key TFs (*STAT1*, *NR3C1*, *CREM*) (**Supplementary Figures S3F, G**), with the *FKBP4*/*NR3C1*/*NRF2* axis known to promote DC maturation (42). Meanwhile, both macrophages and monocytes demonstrated significant communication with CD8_Tex cells (**Figure 4H**).

To spatially characterize the cellular communication networks identified in our single-cell analyses, we performed spatial transcriptomic profiling using canonical markers for cDC2s (*FCER1G*, *CD1C*), macrophages (*CD68*, *APOC1*), and T cell subsets. Our analysis revealed fundamentally distinct organizational patterns between HPV-positive and HPV-negative tumors. In HPV-positive carcinomas, we observed significant spatial co-localization between cDC2-enriched regions and CD4^+^ T cell zones. In HPV-negative patients, the regions with high expression of myeloid cell markers closely overlap with those of CD8^+^ T-cell markers (**Figure 4I**). These findings suggest distinct cell-cell communication patterns between HPV-positive and HPV-negative tumors. Antigen presentation was predominantly carried out by cDC2s through MHC II, and markedly stronger antitumor immune responses were triggered in a CD4^+^ T cell-dependent manner in HPV-positive tumors.

### HPV infection-Dependent Epithelial cell crosstalk and immune pathways analysis

**Figure 5:**
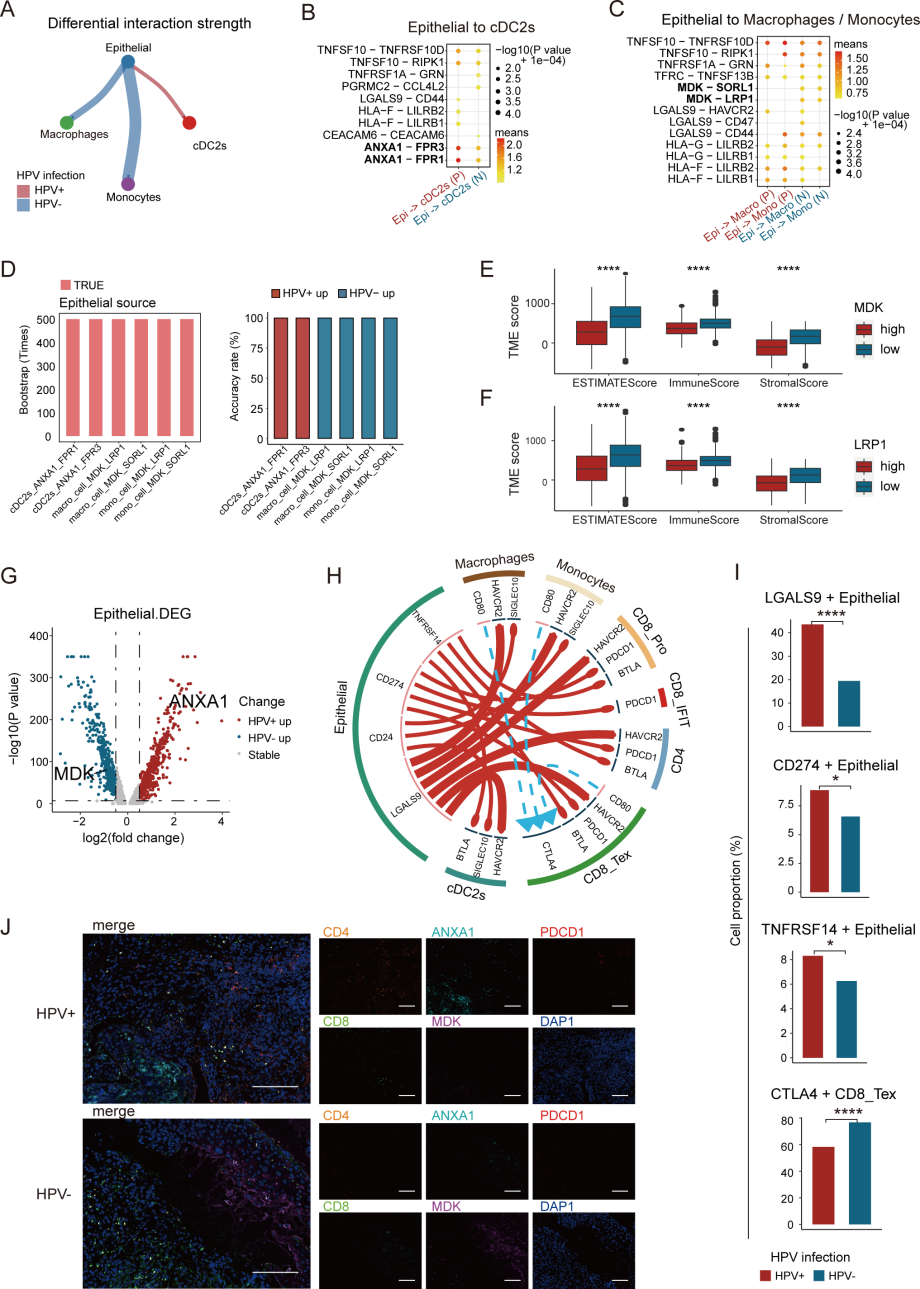
Analysis of epithelial cell-cell crosstalk in HPV-positive and HPV-negative samples. **(A)** Difference in interaction strength between epithelial cells and cDC2s, as well as monocytes and macrophages in HPV-positive and HPV-negative samples, where red and blue indicate high expression in HPV-positive and HPV-negative samples, respectively. **(B)** Potential ligand-receptor pairs between epithelial cells and cDC2s in HPV-positive and HPV-negative CC (P, HPV-positive; N, HPV-negative). **(C)** Potential ligand-receptor pairs between epithelial cells and monocytes and macrophages in HPV-positive and HPV-negative CC. **(D)** Bar graph showing significant differences in ligand-receptor pairs among the 500 bootstrap samples. **(E, F)** High expression of *MDK* and LRP1 was significantly associated with poor immune infiltration. **(G)** Volcano plot showing the changes in gene expression in epithelial cells of HPV-positive group and HPV-negative group, where red represents genes with higher expression in HPV-positive CC and blue represents genes with higher expression in HPV-negative CC. If -log10 (p-value) equals Inf, the maximum value of -log10 (p-value) is set to 350 (Wilcoxon test, p = 0.0000001, logFC.threshold = 0.5). **(H)** Potential cell-cell communication networks in HPV-positive and HPV-negative samples. The thickness of the lines represents the relative expression of ligands, where red and blue indicate high expression in HPV-positive and HPV-negative samples, respectively. The size of the arrows represents the relative expression of receptors. **(I)** Expression proportion of *LGALS9*, *CD274*, *TNFRSF14* gene between HPV-positive and HPV-negative epithelial cells, and the expression proportion of *CTLA4* gene between HPV-positive and HPV-negative CD8_Tex cells (chi-square test, *p < 0.05, **p < 0.01, ***p < 0.001, ****p < 0.0001). **(J)** Representative immunostaining for *CD8*, *CD4*, *ANXA1*, *MDK* and *PDCD1* showing distinct expression levels in HPV-positive and HPV-negative CC patients. Scale bars, 200 ×.

To explore the potential mechanisms underlying immune cell recruitment, we analyzed the ligand-receptor interactions of chemokines between different cell clusters in HPV-positive and HPV-negative tumors. CellPhoneDB analysis revealed that in HPV-positive tumors, epithelial cells exhibited a stronger interaction with cDC2s (**Figure 5A**), primarily via *ANXA1*-*FPR1* and *ANXA1*-*FPR3* (**Figure 5B**). In contrast, epithelial cells in HPV-negative tumors preferentially interacted with macrophages and monocytes, mainly through *MDK*-*SOLR1* and *MDK*-*LRP1* (**Figure 5C**). We conducted a bootstrapped verification of ligand-receptor interactions, and found significant differences in ligand-receptor pairs in 500 bootstrapped samples (**Figure 5D**). Previous studies have demonstrated that the *MDK*-*LRP1* interaction enhances immunosuppression in gallbladder carcinoma (43), prompting us to investigate whether a similar mechanism exists in CC.

To investigate this possibility, we assessed the impact of *MDK* and *LRP1* expression levels on the TME of CC. Using the ESTIMATE tool (20), we quantified the tumor immune infiltration score. The results showed that higher expression of *MDK* and *LRP1* was associated with reduced immune infiltration. Moreover, the Stromal Score, Immune Score, and ESTIMATE Score were all higher in the low *MDK*/*LRP1* expression group compared to the high-expression group (**Figures 5E, F**). These findings suggest that during CC progression, the overexpression of *MDK* in tumor cells and *LRP1* in macrophages contributes to the establishment of an immunosuppressive environment.

To uncover the transcriptional signatures of tumor cells, we performed differential gene expression analysis on epithelial cells from both HPV-positive and HPV-negative tumors. *ANXA1* was upregulated in HPV-positive tumors, while *MDK* was upregulated in HPV-negative tumors (**Figure 5G**). Functional enrichment analysis showed that HPV-positive tumors were enriched in interferon-related pathways, which are crucial for DC maturation and CD4^+^ T cell development (44), whereas HPV-negative tumors were enriched in amino acid metabolism pathways (**Supplementary Figure S4A**). In HPV-positive tumors, epithelial cells regulated cDC2s through *ANXA1*-*FPR1/3*, which in turn influenced CD4^+^ T cells and CD8^+^ IFIT cells via MHC II and *LGALS9*-*CD45*, enhancing immune activation. In contrast, HPV-negative tumors relied on *MDK*-*LRP1*/*SORL1* signaling to regulate monocytes and macrophages, which interacted with CD8^+^ T cells through MHC I, shaping a distinct immune landscape (**Figure 5C**).

Immune checkpoint analysis revealed that HPV-positive tumors exhibited high expression of *LGALS9*, *CD274*, and *TNFRSF14*, whereas HPV-negative tumors featured *CD80/86*-*CTLA4* interactions, associated with CD8^+^ T cell exhaustion (**Figures 5H, I**). Additionally, experimental validation confirmed that HPV-positive tumors exhibited a higher infiltration of CD4^+^ T cells, along with increased expression of *ANXA1* and programmed cell death protein 1, whereas HPV-negative tumors were enriched in CD8^+^ T cells and displayed elevated *MDK* expression (**Figure 5J**). In summary, these findings suggest that checkpoint blockade therapies may be more effective in HPV-positive tumors but less beneficial for HPV-negative cases. HPV infection profoundly shapes the TME, driving immune regulatory differences and influencing therapeutic responses.

### Construction of an epithelial−related prognostic signature

**Figure 6:**
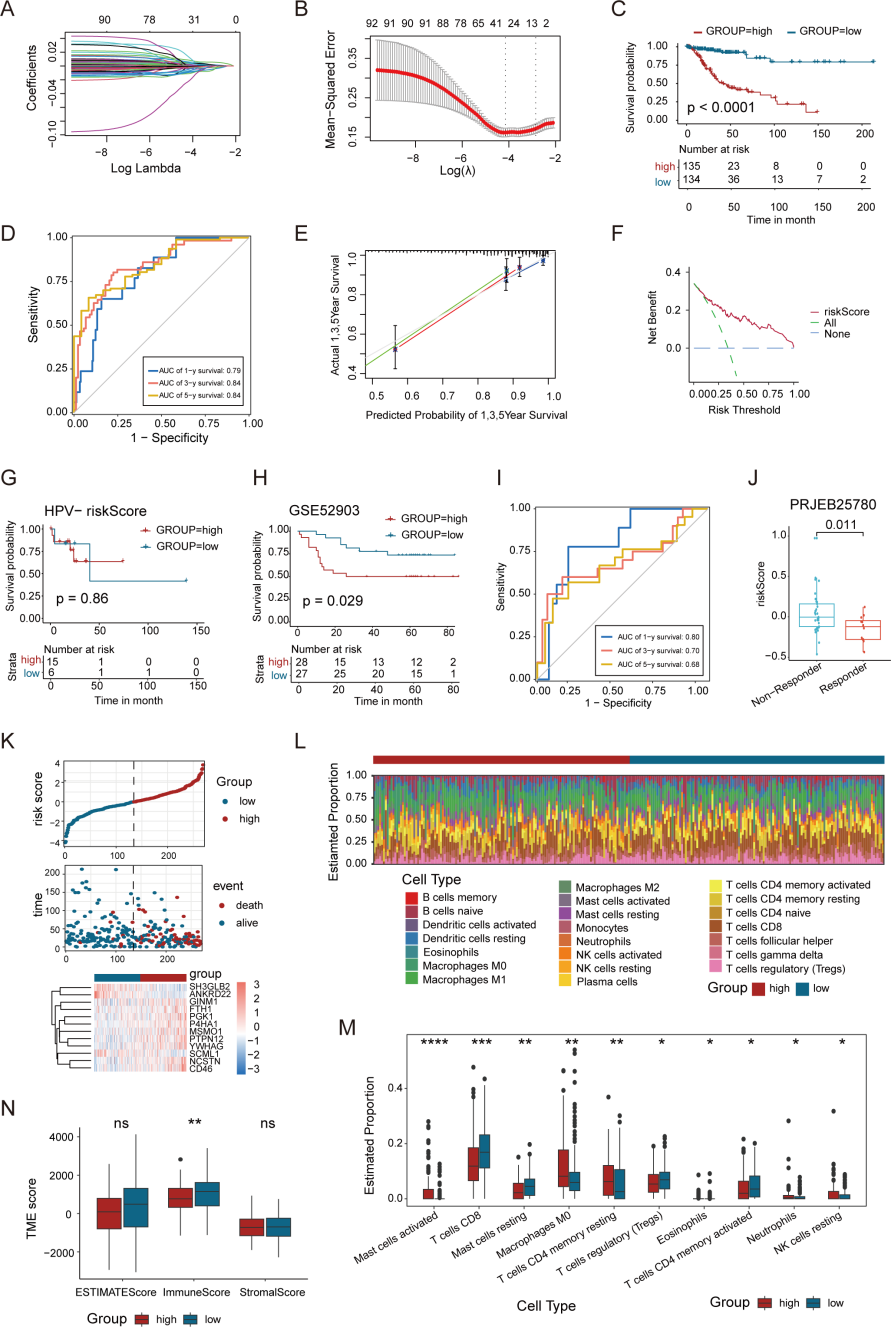
A newly-identified ERS exhibits robust predictive power regarding the prognosis of CC. **(A, B)** LASSO regression was performed on the 92 OS-related genes. Cross-validation was carried out within the LASSO regression model to select the tuning parameter. The abscissa represents the log (λ) value, and the ordinate represents the partial likelihood deviance. The red dots in the figure denote the partial likelihood deviations ± standard error for various tuning parameters. **(C)** Kaplan–Meier curves of OS based on the ERS risk score in the HPV-positive CC patients. **(D)** Time-dependent ROC curve demonstrating the survival accuracy of the model. **(E)** Calibration curves for the risk score. **(F)** Decision curve for the risk score. **(G)** Kaplan–Meier curves of OS based on the ERS risk score in the HPV-negative CC patients. **(H)** Kaplan–Meier curves of the OS of patients in the GSE52903 cohort. **(I)** Time-dependent ROC curves for predicting 1-, 3-, and 5-year OS in the GSE52903 cohort. **(J)** Association between ERS risk scores and immunotherapy response in the PRJEB25780 cohort. **(K)** Distribution of risk score, survival status (red dots indicate dead, blue dots indicate alive) and the gene expression of 12 model genes. **(L)** Stacked bar graph of immune infiltration showed differential expression of immune infiltration in high and low ERS group. **(M)** Box-and-line plot showed the differential immune infiltration of 10 immune cells which had significant differences in high and low ERS groups. **(N)** Box line plot showed the difference between high and low ERS groups in Stromal Score, Immune Score, and ESTIMATE Score. (*p < 0.05, **p < 0.01, ***p < 0.001, ****p < 0.0001).

The molecular and immune landscape of CC varies significantly based on HPV status, leading to distinct prognostic outcomes. However, the impact of epithelial-related transcriptional alterations in this context remains unclear. To address this, we constructed an epithelial-related risk score (ERS) based on DEGs upregulated in epidermal cells in HPV-positive samples. This model was constructed using 269 HPV-positive patients in the TCGA CC cohort as a training set, with 92 prognostic genes identified through univariate COX analysis (*P*<0.05). Further, LASSO analyses and multivariate Cox regression analyses were applied to refine the model, ultimately incorporating 12 key genes (**Figures 6A, B**). We performed permutation-based validation on the 12 ERS genes, confirming the statistical reliability of the ERS gene selection (**Supplementary Figure S4B**). The risk score was calculated based on the regression coefficients derived from univariate Cox analysis.

Patients were then divided into high- and low-risk groups based on the median risk score to evaluate the prognostic value of the ERS. Kaplan-Meier survival analysis showed significant differences in patient overall survival (OS) between the high- and low-risk groups (**Figure 6C**). In addition, ROC curve analysis assessed the predictive accuracy of the ERS, with 1-, 3-, and 5-year AUC values demonstrating strong predictive power of the TCGA training set (**Figure 6D**). The calibration curve validated the consistency between the model’s predicted results and the actual outcomes (**Figure 6E**), while DCA assessed the net benefit of the model in the process of clinical decision-making (**Figure 6F**). We further evaluated the performance of ERS in the HPV-negative subgroup, and the results showed that the ERS risk score did not exhibit prognostic predictive value in patients with HPV-negative CC (*P* = 0.86) (**Figure 6G**). This finding indicates that the prognostic utility of the ERS model is specific to the HPV-positive subgroup. We validated the ERS risk model in an independent dataset GSE52903, which included survival prognosis data from 55 HPV-positive patients. The results of survival analysis were well validated in the GSE52903 cohort. Meanwhile, the ROC curve assessment of the model demonstrated that it had a favorable predictive performance for the prognosis of patients in GSE52903 (**Figures 6H, I**). Additionally, we observed that in patients with metastatic gastric cancer receiving pembrolizumab treatment (45), the ERS risk scores were significantly enriched in non-responders (**Figure 6J**), indicating that the ERS risk model can predict the efficacy of immunotherapy.

Further analysis of the relationship between risk scores, survival time, and survival status highlighted that patients in the high-risk group had a poorer prognosis. The heatmap illustrated the relationship between 12 genes and risk levels, with a negative correlation between the high-risk group and genes such as *YWHAG*, *SH3GLB2*, *ANKRD22*, *SCML1*, and *P4HA1* (**Figure 6K**). The correlation between risk scores and immune cell infiltration in CC was examined. Immune cell infiltration was significantly lower in the high-risk group compared to the low-risk group, with higher levels of resting CD4^+^ T cells and CD8^+^ T cells in the low-risk group. In contrast, Tregs and M0 macrophages were notably more abundant in the high-risk group (**Figures 6L, M**). The Stromal Score, Immune Score and ESTIMATE Score were compared between the high and low ERS score groups, revealing no significant difference in the Stromal Score and ESTIMATE Score. However, the Immune Score was higher in the low ERS score group than in the high ERS score group (**Figure 6N**). This comprehensive analysis emphasizes the potential of ERS in predicting the clinical prognosis and immunotherapy response of CC patients, providing new insights for future research on CC treatment.

## Discussion

HPV-negative CC accounts for approximately 3-8% of all CC cases and demonstrates significantly poorer clinical outcomes. Compared to HPV-positive CC, along with distinct molecular profiles. Despite these differences, current clinical guidelines maintain similar treatment recommendations for both subtypes (3, 4), highlighting an urgent need for dedicated research into novel targeted therapies for HPV-negative CC. While bulk RNA-sequencing studies have identified HPV status-dependent alterations in gene expression patterns and immune cell composition (46–48), these single-omics approaches lack the cellular resolution necessary to fully characterize tumor heterogeneity. Recent therapeutic advances in CC have emphasized the importance of considering TME dynamics and immune infiltration patterns (1, 49). Since TME-mediated immune suppression contributes to metastatic progression and treatment resistance (50), understanding HPV-specific TME modifications is critical. Our study comprehensively characterized the variants of epithelial and immune cell clusters at the single-cell and ST level, providing novel insights into the remodeling of the tumor ecosystem in CC based on HPV infection status. These findings establish a foundation for developing more effective treatment strategies to achieve improved long-term disease control.

The ST technology empowers the in-depth characterization of the spatial patterns of gene expression within tissues. It not only serves as a crucial tool for exploring the intricate mechanisms of intercellular communication but also paves the way for mapping the spatiotemporal sequence of cell development. However, current ST platforms face technical constraints, being limited by either suboptimal single-cell resolution or incomplete transcriptome coverage. The innovative iStar technology overcomes these limitations by integrating machine learning with ST, providing rapid and precise cellular deconvolution of tissue microenvironments. This breakthrough facilitates near single-cell resolution ST data generation with full transcriptome coverage. In our research, we harnessed the iStar technology to conduct a meticulous analysis of untreated CC tumors. As a result, we were able to furnish a spatially resolved and exquisitely detailed map of the heterogeneity landscape within CC tumors. Spatial mapping revealed that memory B cells and naive B cells, along with CD4^+^ T cells and DCs, exhibited adjacent distributions within the CC. Naive B cells mark an early stage in the developmental trajectory of B cells. In contrast, memory B cells are a distinct cell type endowed with memory function, which are generated subsequent to B-cell engagement in an immune response. The close-proximity arrangement of these cell populations in the TME confers a distinct advantage, facilitating the acquisition of differentiation signals by naive B cells. This, in turn, promotes their transition into memory B cells (24). This coordinated cellular network participates in tumor immune surveillance, forming a critical component of anti-tumor immunity (27). DCs stand as the most potent antigen-presenting cells (APCs) in the human body. Notably, once DCs internalize tumor antigens, they can efficiently relay antigenic information to neighboring CD4^+^ T cells, thereby instigating the activation process of CD4^+^ T cells (51). Specifically, DCs initiate a maturation cascade. This maturation event is characterized by an upregulation in the expression of co-stimulatory molecules, such as *CD80* and *CD86*, on their cell surface. These co-stimulatory molecules then engage in molecular interactions with counterparts like *CD28* on the surface of CD4^+^ T cells, furnishing the essential second signal requisite for the complete activation of T cells (25). Our integrated spatial atlas elaborately delineates the cell composition and gene expression in the anatomical regions of CC tissue. This, in turn, equips us with the means to investigate the complex TME dynamics of human CC under diverse HPV statuses.

Our study revealed substantial disparities in both cellular composition and transcriptomic profiles between HPV-negative and HPV-positive CC specimens. A particularly striking observation was the significantly elevated frequency of CD4^+^ T cells, cDC2s cells, and interferon-related CD8^+^ T cells subsets in HPV-positive tumors, whereas HPV-negative specimens exhibited predominance of distinct CD8^+^ T cell subpopulations. This distribution suggests different immune response dynamics between HPV-positive and HPV-negative CC, despite the critical role of CD8^+^ T cells in controlling viral infections. Analysis of the TCGA data (including 21 HPV-negative cases among 290 CC patients) provided partial validation of these findings. For the first time, our study indicates that HPV-positive CC may exhibit stronger immune responses mediated by CD4^+^ T cells, with cDC2s playing a crucial role in antigen presentation. Conversely, HPV-negative CC tends to evoke robust immune responses mediated by CD8^+^ T cells, significantly supported by monocytes in antigen presentation (52). DCs, as key a APCs, bridge the innate and adaptive immune systems (51). The interaction between T-cell receptors and peptide epitopes from tumor-associated and tumor-specific antigens bound to MHC on the surface of APCs, including DCs and macrophages, initiates and modulates the immune response against HPV-infected cells, ultimately influencing tumor progression (53). HPV serves as a necessary, albeit insufficient, etiological factor in CC development (2). Previous research on HPV-positive head and neck squamous cell carcinomas has demonstrated a more favorable prognosis and higher radiosensitivity compared to their HPV-negative, radioresistant counterparts (54).

The dynamic crosstalk between epithelial and immune cells plays a pivotal role in malignant tumor pathogenesis (55). Our analysis revealed fundamentally distinct epithelial-immune interaction networks in HPV-positive versus HPV-negative CC. In HPV-positive tumors, epithelial cells functioned as master regulators of cDC2s through *ANXA1*-*FPR1/3* signaling. These activated cDC2 then orchestrated immune responses by modulating CD4^+^ T cells and via MHC class II molecules and interferon-responsive CD8^+^ T cell subsets through *LGALS9*-*CD45* interactions. Several studies have emphasized the relationship between FPR1 and DC maturation and activation (56). Specifically, the interaction between *ANXA1* and *FPR1* in DCs enhances anti-tumor immunity by promoting the phagocytic uptake of dead cell antigens by DCs (57, 58). These findings highlight the potential of targeting the *ANXA1*-*FPR1* axis as an immunotherapeutic approach. In contrast, HPV-negative tumors exhibited predominant epithelial-monocyte/macrophage communication via *MDK*-*LRP1*/*SORL1* signaling, with subsequent MHC class I-mediated regulation of CD8^+^ T cells. Studies have demonstrated that *MDK* secreted by tumor cells binds to the *LRP1* receptor on the surface of macrophages, polarizing macrophages toward the M2 phenotype (59, 60) and promoting the secretion of the cytokine *CXCL1*. In turn, *CXCL1* further recruits immunosuppressive cells and tumor-associated macrophages to support tumor cell growth (60). Zhang et al. reported that in gallbladder cancer, *MDK* interacts with the receptor *LRP1*, promoting the differentiation of immunosuppressive macrophages. This ultimately reduces anti-cancer immunity and contributes to cancer development (43). Consistently, we observed significant overexpression of tumor-associated ligand-receptor pair *MDK-LRP1* in CC, correlating with diminished immune cell infiltration. Through high-resolution spatial mapping using the BayesSpace algorithm (9), we found that in HPV-positive CC, cDC2s are in closer proximity to CD4^+^ T cells. Conversely, in HPV-negative CC, macrophages are in closer proximity to CD8^+^ T cells. Therefore, HPV appears to influence the TME of CC, potentially regulating the composition of immune cells, cell-cell interactions, and diverse immune pathways. This regulation can, to some extent, enhance the anti-tumor response. Notably, due to the relatively small sample size of the scRNA-seq and ST dataset, the subtype-specific immune interaction patterns identified in this study should be interpreted with caution. Although previous studies support the robustness of these observations in our cohort, their generalizability to larger and more diverse patient populations may be limited. Future studies with larger sample sizes are warranted to further validate and refine these interaction signatures.

Immunotherapy has become a promising strategy in the fight against cancer, leveraging host immune system modulation to achieve precise tumor targeting (61). This approach offers the potential for improved treatment outcomes and extended survival for patients with CC (1, 62). Pembrolizumab currently represents the sole FDA-approved immune checkpoint inhibitor for CC, with its application being especially warranted in HPV-driven cases exhibiting programmed cell death 1 ligand 1 (*PD-L1*) or cytotoxic t-lymphocyte associated protein 4 (*CTLA-4*) expression (1). *PD1*/*PD-L1* blockade primarily functions by preventing CD8^+^ cell exhaustion, while *CTLA-4* inhibitors work by activating new T cells and suppressing Tregs (49, 62). However, HPV-negative CC—characterized by an immunologically quiescent (“cold”) TME mdash;typically does not respond to traditional immunotherapy. Consequently, ongoing research is exploring innovative immunotherapeutic approaches to expand treatment options and enhance patient outcomes. HPV-associated CCs often exhibit upregulated *PD-L1* expression, and *PD-L1*/*PD1* inhibitors have shown promising response rates in CC (63). While blocking the *PD1*/*PD-L1* axis has proven effective in disrupting tumor immune tolerance across various cancers, high *PD-L1* expression has also been linked to increased tumor-infiltrating lymphocyte levels and poorer survival in HPV-independent CC (64–66). By elucidating the unique molecular mechanisms underlying tumorigenesis in these patients, new or combined immunotherapeutic strategies may be developed, offering significant clinical benefits.

Our study revealed distinct immune checkpoint profiles between HPV-associated and HPV-independent cervical carcinomas. HPV-positive CC tumors exhibited epithelial cells with significant enrichment of immune checkpoint ligands (*LGALS9*, *CD274*, and *TNFRSF14*), whereas HPV-negative carcinomas predominantly activated the *CD80/86*-*CTLA4* pathway. *CTLA-4* and *CD28* share the ligands *CD80* and *CD86*, but *CTLA-4* binds with significantly higher affinity, leading to immune response deactivation (67, 68). *CTLA-4* blockade exerts dual antitumor effects by (1) Enhancing T-cell cytotoxicity through immune checkpoint disruption and (2) Directly inhibiting FOXP3+ Tregs function (69). Mechanistically, while the *PD1/PD-L1* primarily regulates effector-phase immunity, the *CTLA-4/CD86* pathway governs early T-cells priming and activation of naïve/memory T cell populations (70). Targeting *CTLA-4* thus holds significant promise for improving immunotherapy efficacy in HPV-negative CC. Exploring novel or combined immunotherapeutic options could provide valuable insights and opportunities for enhancing treatment outcomes in HPV-positive CC. In conclusion, these findings provide groundbreaking insights into the unique immunological landscapes of different CC subtypes based on HPV status. This study identifies potential avenues for personalized therapeutic strategies by leveraging distinct immune checkpoint profiles, particularly for HPV-negative CC.

In view of the pivotal role that epithelial cells play in the occurrence and development of CC, we selected the DEGs of epithelial cells in HPV-positive and HPV-negative CC to construct a prognostic model. From the perspective of cell communication, epithelial cells are located upstream in the cell communication network. They can regulate the functions of downstream immune cells such as DCs and macrophages by expressing a variety of receptors. In HPV-positive CC patients, the abnormal state of epithelial cells may lead to a disorder in their regulatory function on downstream immune cells, thereby affecting the patients’ immune response and prognosis. Therefore, investigating the DEGs of epithelial cells contributes to revealing the potential mechanisms by which they regulate the tumor immune microenvironment, providing important clues for predicting the prognosis of HPV-positive patients. Moreover, epithelial cells are one of the major cell types in the TME of CC. This characteristic endows them with a central position in tumor development and the formation of intratumoral heterogeneity. By analyzing the DEGs of epithelial cells, we can gain a deeper understanding of the nature of intratumoral heterogeneity and uncover molecular markers closely related to the prognosis of HPV-positive patients. The ERS performed well in the TCGA cohorts. The AUC values at 1-year, 3-year, and 5-year time points all exceeded 0.79. Furthermore, the infiltration of immune cells in the low-risk group was significantly higher than that in the high-risk group, indicating that ERS may be helpful in evaluating the response to immunotherapy. Our study in the PRJEB25780 cohort confirmed the correlation between ERS and the efficacy of immunotherapy. ERS can assist us in better understanding the pathogenesis and biological behavior of CC, thus holding potential clinical application value.

This study is constrained by three primary limitations. Firstly, the small sample size in scRNA and ST analysis limits the statistical power and generalizability of our findings. A key reason for this is the low clinical incidence of HPV-negative CC, which makes sample accumulation challenging. The exclusion of certain cell types, such as B cells and fibroblasts, due to insufficient sample quantity, results in a relatively small representation of these clusters in our analysis. Further investigation is needed to elucidate their roles in the TME and their interactions with other cells. Larger sample sizes in future studies are crucial to validate our conclusions and allow for a more in-depth exploration of cell subclusters associated with treatment response. Secondly, although ST has provided valuable insights, the relatively low spatial resolution of the Visium platform may not fully capture the intricate cellular interactions and heterogeneity within cervical tumors. In the future, an integrated multi-omics approach that combines sub-cellular resolution ST with proteomics and metabolomics could offer a more comprehensive understanding of the HPV-related immune microenvironment and prognostic features in CC. Thirdly, variability in tumor volume in CC can significantly impact cell proportions and heterogeneity. Biopsy examination alone may not capture the entire tumor, and additional multipoint inspections or resections could help mitigate this limitation.

## Conclusions

Collectively, our study underscored the molecular heterogeneity within the TME based on the HPV infection status and comprehensively characterized the variants of epithelial and immune cell clusters at the single-cell and ST levels, providing novel insights into the remodeling of the tumor ecosystem in CC based on HPV infection status. Lastly, the differential genes of epithelial cells were utilized to construct the ERS, a prognostic prediction model for CC. These findings may provide insights for the development of new treatment strategies for CC.

## Data Availability

The single cell RNA sequencing data presented in the study are deposited in the GEO repository, accession number GSE171894. The spatial transcriptomics data presented in the study are deposited in the Zenodo data repository, accession number 10.5281/zenodo.16917924.

## Supplementary material

Supplementary Figure 1 Seurat-based UMAP visualization of cell clusters. **(A)** Feature plots of canonical markers across five cell types. **(B)** Violin diagram showing expression of eight major cell phenotypic marker genes.

Supplementary Figure 2 Single-cell landscape of HPV-positive and HPV-negative CC. **(A)** Feature plots of canonical markers across seven cell types. **(B)** Heatmap showing expression of marker genes for the seven major cell phenotypes. **(C)** Tissue preference of each cluster measured by Ro/e. **(D)** Heatmap showing expression of marker genes for the nine T/NK cell phenotypes. **(E)** UMAP visualization of T/NK cells, colored by HPV infection status. **(F)** UMAP plots of T and NK cells, colored by cell types (left). T and NK cell cluster frequency comparing HPV-positive and HPV-negative samples (middle). Fraction of T and NK cells for HPV-positive and HPV-negative samples (right, chi-square test, *p < 0.05). **(G)** Proportion of T and NK cells in naïve CD4^+^ T, Treg CD4^+^ T, exhausted CD8^+^ T, memory CD8^+^ T, interferon-related CD8^+^ T, proliferating CD8^+^ T and NK cells between HPV-positive and HPV-negative CC (chi-square test, *p < 0.05). **(H)** Heatmap showing expression of marker genes for the five Myeloid cell phenotypes. **(I)** UMAP visualization of Myeloid cells, colored by HPV infection status (left); Fraction of Myeloid cells in monocytes and cDC2s cells for HPV-positive and HPV-negative samples (right, chi-square test, *p < 0.05).

Supplementary Figure 3 Distinct developmental trajectory inference between HPV-positive and HPV-negative CC. **(A)** Distribution of CD4 T subpopulations during the transition along the pseudotime. **(B)** Distribution of HPV-positive (left) and HPV-negative (right) CD4 T cell subpopulations during the transition along the cell type. **(C)** Differential expression of *BHLHE40*, *RBPJ*, *SATB1*, and *TRIM22* in HPV-positive and HPV-negative CD8^+^ T cells, as well as between the fate1 and fate2 trajectories. Solid lines represent the fate1 trajectory, and dashed lines represent the fate2 trajectory, with red indicating HPV-positive samples and blue indicating HPV-negative CC. **(D)** Volcano plot depicting gene expression changes in cDC2s cells between HPV-positive and HPV-negative groups. Red dots represent genes with higher expression in the HPV-positive group, while blue dots indicate higher expression in the HPV-negative group (Wilcoxon test, p = 0.0000001, logFC threshold = 0.5). **(E)** Violin plot illustrating the expression of MHC II molecules and dendritic cell maturity scores in cDC2s cells between HPV-positive and HPV-negative samples, with colors representing the mean values (Wilcoxon test). **(F)** Scatter plot displaying the specificity scores of regulons in cDC2s cells, highlighting the top 5 regulons. **(G)** Violin plot showing the expression of the transcription factor NR3C1 in cDC2s cells in HPV-positive and HPV-negative CC, with colors representing the mean expression level (Wilcoxon test).

Supplementary Figure 4 Pathway enrichment analysis of HPV-positive/negative CC and permutation-based validation of ERS genes. **(A)** DEG pathway enrichment plots for epithelial cells in HPV-positive and HPV-negative groups using ReactomePA. Pathways are sorted by count, with the left plot showing pathways enriched in HPV-positive CC and the right plot showing pathways enriched in HPV-negative CC. DEG, differentially expressed gene. **(B)** Histogram showing permutation-based verification of 12 ERS genes.
