# Supplementary figures and images for: Integrated multi-omics analysis of single-cell and spatial transcriptomics reveals distinct hpv-associated immune microenvironment features and prognostic signatures in cervical cancer
Source: Front Immunol. 2025 Sep 16;16:1612623. doi: 10.3389/fimmu.2025.1612623 (PMC12481161; doi:10.3389/fimmu.2025.1612623)

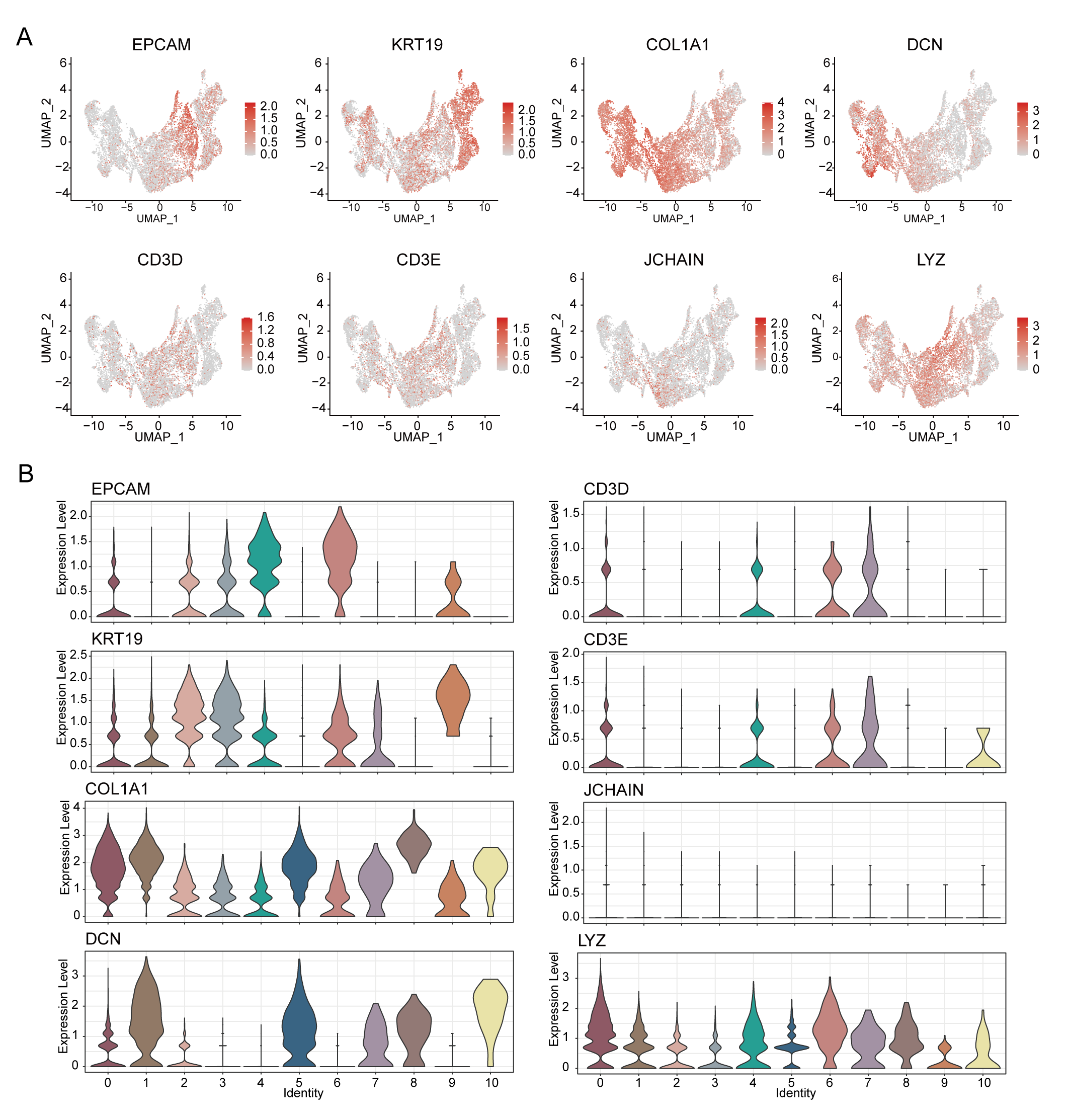

Supplement: Supplementary Figure 1 — Seurat-based UMAP visualization of cell clusters. (A) Feature plots of canonical markers across five cell types. (B) Violin diagram showing expression of eight major cell phenotypic marker genes. [file Image1.tiff]

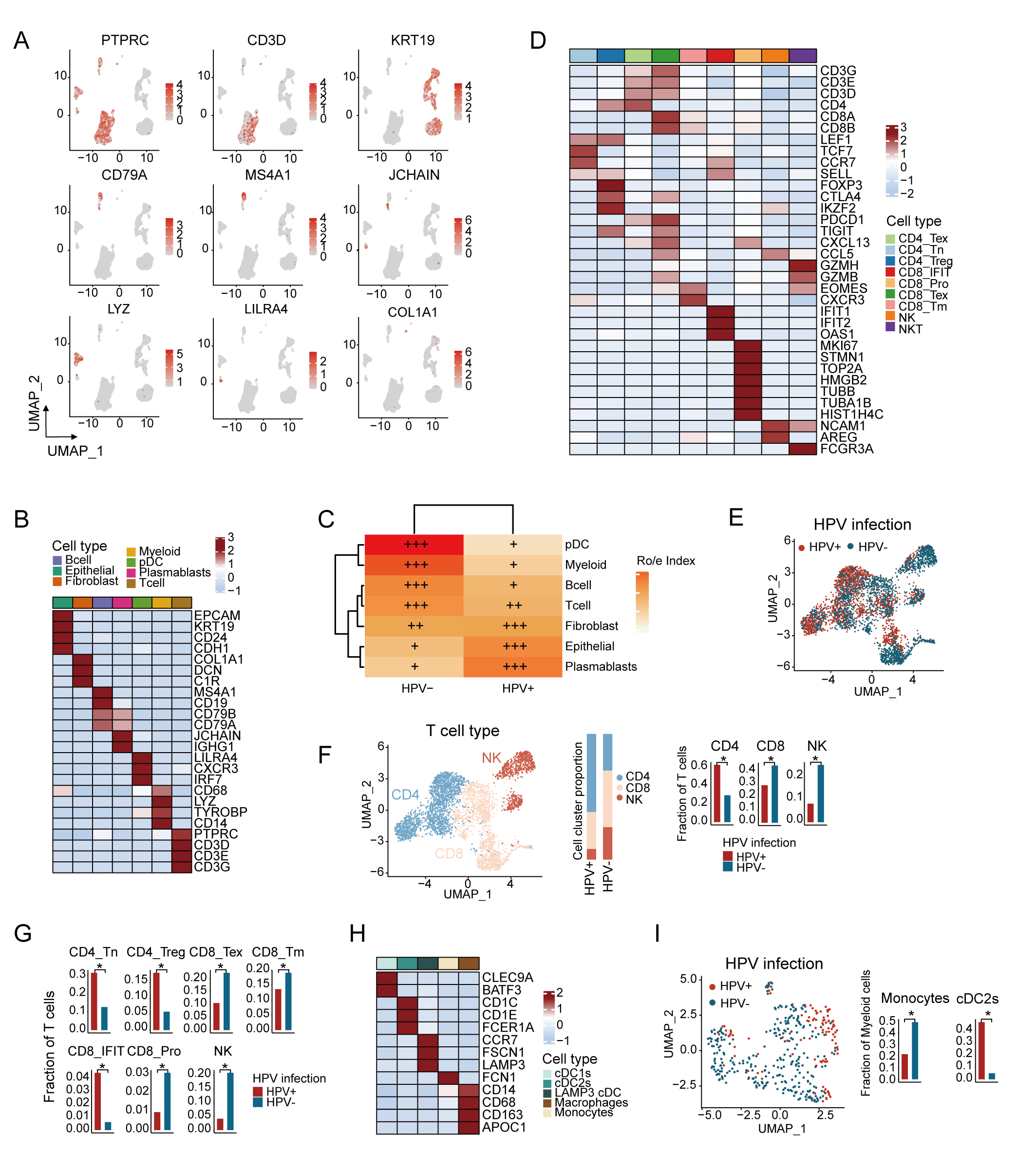

Supplement: Supplementary Figure 2 — Single-cell landscape of HPV-positive and HPV-negative CC. (A) Feature plots of canonical markers across seven cell types. (B) Heatmap showing expression of marker genes for the seven major cell phenotypes. (C) Tissue preference of each cluster measured by Ro/e. (D) Heatmap showing expression of marker genes for the nine T/NK cell phenotypes. (E) UMAP visualization of T/NK cells, colored by HPV infection status. (F) UMAP plots of T and NK cells, colored by cell types (left). T and NK cell cluster frequency comparing HPV-positive and HPV-negative samples (middle). Fraction of T and NK cells for HPV-positive and HPV-negative samples (right, chi-square test, *p < 0.05). (G) Proportion of T and NK cells in naïve CD4+ T, Treg CD4+ T, exhausted CD8+ T, memory CD8+ T, interferon-related CD8+ T, proliferating CD8+ T and NK cells between HPV-positive and HPV-negative CC (chi-square test, *p < 0.05). (H) Heatmap showing expression of marker genes for the five Myeloid cell phenotypes. (I) UMAP visualization of Myeloid cells, colored by HPV infection status (left); Fraction of Myeloid cells in monocytes and cDC2s cells for HPV-positive and HPV-negative samples (right, chi-square test, *p < 0.05). [file Image2.tiff]

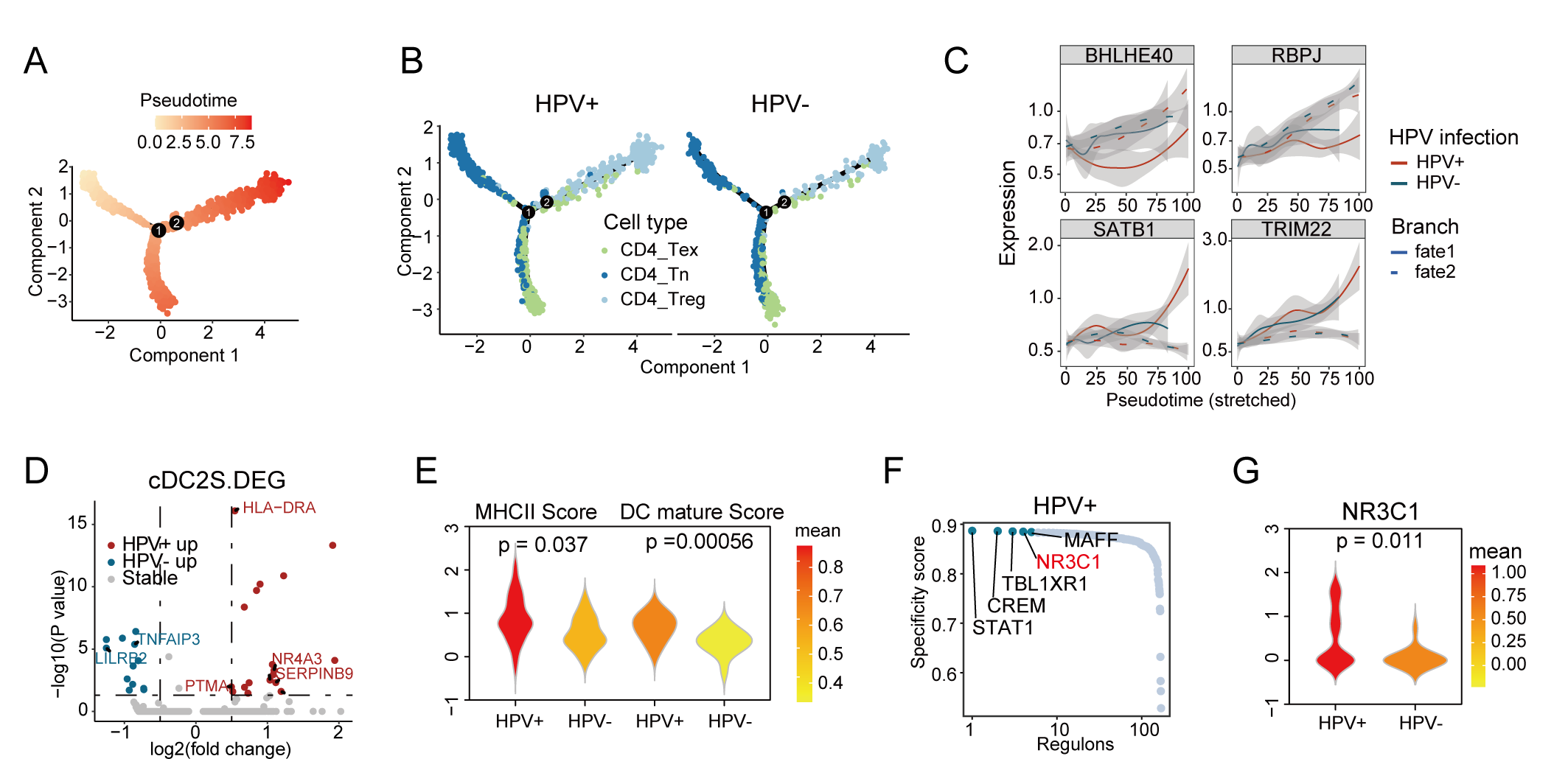

Supplement: Supplementary Figure 3 — Distinct developmental trajectory inference between HPV-positive and HPV-negative CC. (A) Distribution of CD4 T subpopulations during the transition along the pseudotime. (B) Distribution of HPV-positive (left) and HPV-negative (right) CD4 T cell subpopulations during the transition along the cell type. (C) Differential expression of BHLHE40, RBPJ, SATB1, and TRIM22 in HPV-positive and HPV-negative CD8+ T cells, as well as between the fate1 and fate2 trajectories. Solid lines represent the fate1 trajectory, and dashed lines represent the fate2 trajectory, with red indicating HPV-positive samples and blue indicating HPV-negative CC. (D) Volcano plot depicting gene expression changes in cDC2s cells between HPV-positive and HPV-negative groups. Red dots represent genes with higher expression in the HPV-positive group, while blue dots indicate higher expression in the HPV-negative group (Wilcoxon test, p = 0.0000001, logFC threshold = 0.5). (E) Violin plot illustrating the expression of MHC II molecules and dendritic cell maturity scores in cDC2s cells between HPV-positive and HPV-negative samples, with colors representing the mean values (Wilcoxon test). (F) Scatter plot displaying the specificity scores of regulons in cDC2s cells, highlighting the top 5 regulons. (G) Violin plot showing the expression of the transcription factor NR3C1 in cDC2s cells in HPV-positive and HPV-negative CC, with colors representing the mean expression level (Wilcoxon test). [file Image3.tiff]

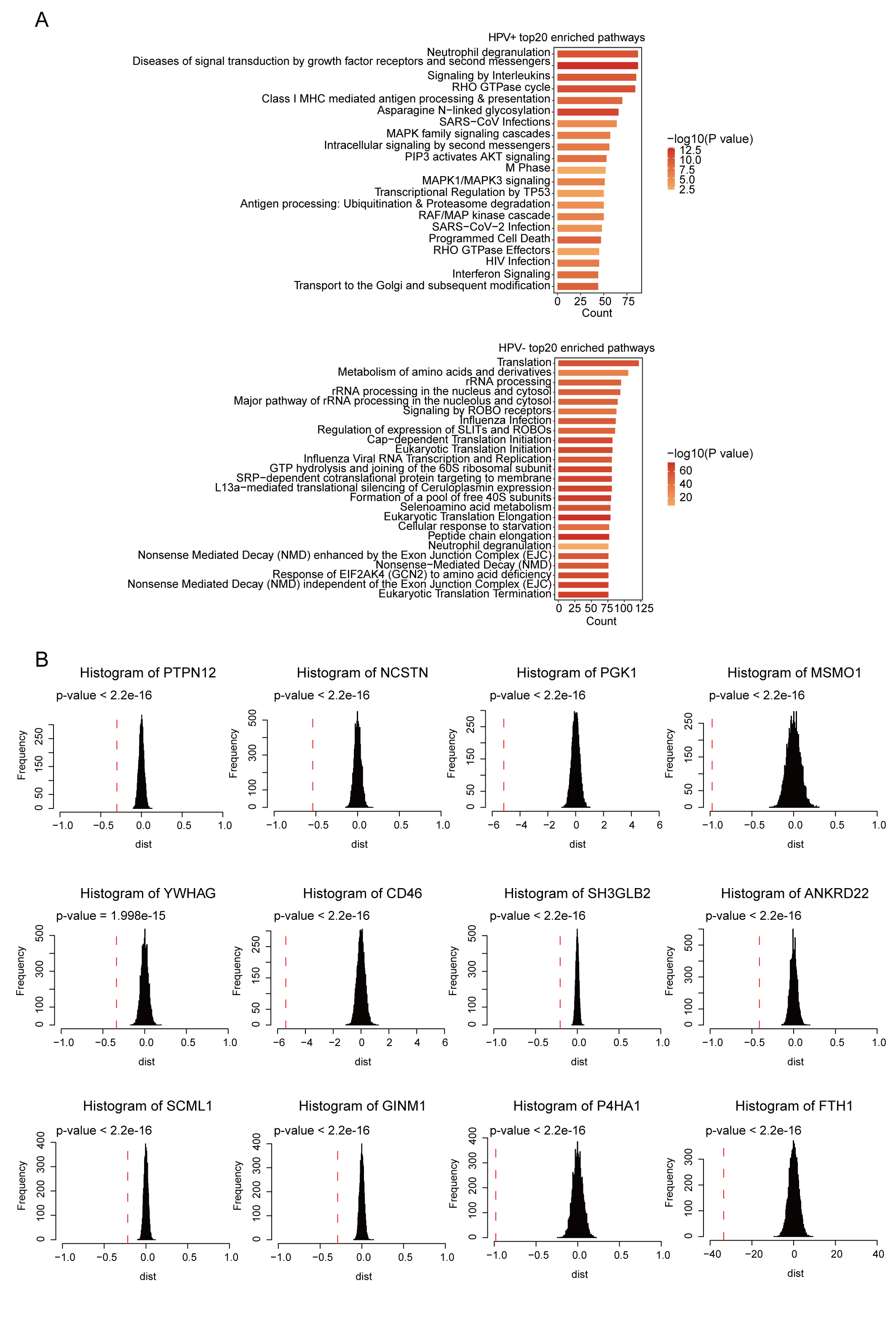

Supplement: Supplementary Figure 4 — Pathway enrichment analysis of HPV-positive/negative CC and permutation-based validation of ERS genes. (A) DEG pathway enrichment plots for epithelial cells in HPV-positive and HPV-negative groups using ReactomePA. Pathways are sorted by count, with the left plot showing pathways enriched in HPV-positive CC and the right plot showing pathways enriched in HPV-negative CC. DEG, differentially expressed gene. (B) Histogram showing permutation-based verification of 12 ERS genes. [file Image4.tiff]
