# Supplementary material for: Integrated multi-omics analysis of single-cell and spatial transcriptomics reveals distinct hpv-associated immune microenvironment features and prognostic signatures in cervical cancer
Source: Front Immunol. 2025 Sep 16;16:1612623. doi: 10.3389/fimmu.2025.1612623 (PMC12481161; doi:10.3389/fimmu.2025.1612623)
Supplement: Supplementary file 5 [file Table1.docx]

**Supplementary Table S1. Clinical characteristics of samples**

| Patient | HPV^*^ | Age | Histopathology |
| --- | --- | --- | --- |
| Patient1 | - | 76 | Squamous cell carcinoma |
| Patient2 | - | 60 | Squamous cell carcinoma |
| Patient3 | + | 71 | Squamous cell carcinoma |
| Patient4 | + | 58 | Squamous cell carcinoma |
| Patient5 | - | 37 | Adenocarcinoma |
| Patient6 | + | 38 | Squamous cell carcinoma |
| Patient7 | + | 40 | Squamous cell carcinoma |

**Supplementary Table S2. Gene lists of MHC class II and dendritic cell maturation.**

| MHCII | HLA-DRA,HLA-DQB2,HLA-DQA2,HLA-DPB1, HLA-DOB,HLA-DMB |
| --- | --- |
| DC maturation | HLA-DRA,HLA-DQB2,HLA-DQA2,HLA-DPB1, HLA-DOB,HLA-DMB,CD80,CD86,CD83,BATF3,SERPINB9,NR4A3,PTMA,CEBPB,PRDM1,NR3C1 |

**Supplementary Table S3. Proportions of Different Cell Clusters in Four Patients**

|  | Bcell | | Epithelial | Fibroblast | Myeloid | pDC | Plasmablasts | Tcell | |
| --- | --- | --- | --- | --- | --- | --- | --- | --- | --- |
| Patient1 | 0.0748 | | 0.1797 | 0.0061 | 0.1298 | 0.0438 | 0.0071 | 0.5586 | |
| Patient2 | 0.0157 | | 0.4359 | 0.0030 | 0.0157 | 0.0056 | 0.0025 | 0.5217 | |
| Patient3 | 0.0220 | | 0.7081 | 0.0031 | 0.0223 | 0.0024 | 0.0040 | 0.2383 | |
| Patient4 | 0.0401 | | 0.2171 | 0.0080 | 0.0210 | 0.0068 | 0.0284 | 0.6786 | |
| **Supplementary Table S4. Gene markers of cell types** | | | | | | | | | |
| Celltype | | | subtypes | | markers | | | | |
|  | | | B cell | | *CD79A*, *CD79B*, *MS4A1*, *CD19* | | | | |
|  |  |  | Plasma cells | | *JCHAIN, IGHG1* | | | | |
|  |  |  | T cell | | *PTPRC, CD3D, CD3E, CD3G* | | | | |
|  |  |  | Myeloid | | *CD68, LYZ, TYROBP, CD14* | | | | |
|  |  |  | pDC | | *LILRA4, CXCR3, IRF7, SPIB, MPEG1* | | | | |
|  |  |  | Fibroblast | | *COL1A1, DCN, C1R* | | | | |
|  |  |  | Epithelial | | *EPCAM, KRT19, CD24, CDH1* | | | | |
| T cell | | | NK cell | | *NCAM1, AREG* | | | | |
|  |  |  | NKT cell | | *NCAM1, FCGR3A* | | | | |
|  |  |  | CD4_Treg | | *CD4, FOXP3, CTLA4, IKZF2* | | | | |
|  |  |  | CD4_Tex | | *CD4, PDCD1, CTLA4, TIGIT, CXCL13* | | | | |
|  |  |  | CD4_Tn | | *CD4, LEF1, TCF7, CCR7, SELL* | | | | |
|  |  |  | CD8_IFIT | | *CD8A, CD8B, IFIT1, IFIT2, OAS1* | | | | |
|  |  |  | CD8_Tex | | *CD8A, CD8B, CTLA4, TIGIT, CXCL13, CCL5, GZMH, GZMB* | | | | |
|  |  |  | CD8_Tm | | *CD8A, CD8B, CCL5, EOMES, SELL,* CCR7, *CXCR3* | | | | |
|  |  |  | CD8_Pro | | *CD8A, CD8B, MKI67, STMN1, TOP2A, HMGB2, TUBB, TUBA1B* | | | | |
| Myeloid cell | | | cDC1s | | *CLEC9A, BATF3, CADM1* | | | | |
|  |  |  | cDC2s | | *CD1C, CD1E, FCER1A* | | | | |
|  |  |  | LAMP3 cDC | | *CCR7, FSCN1, LAMP3* | | | | |
|  |  |  | Macrophages | | *CD68, CD163, APOC1* | | | | |
|  |  |  | Monocytes | | *CD14, FCN1* | | | | |
| CD4_Treg: Treg CD4 T; CD4_Tex: exhausted CD4 T; naïve CD4 T: CD4_Tn; CD8_IFIT: interferon-related CD8 T; CD8_Tex: exhausted CD8 T; CD8_Tm: memory CD8 T; CD8_Pro: proliferating CD8 T. | | | | | | | | | |
